# A ‘Mini Linguistic State Examination’ to classify primary progressive aphasia

**DOI:** 10.1093/braincomms/fcab299

**Published:** 2021-12-21

**Authors:** Nikil Patel, Katie A. Peterson, Ruth U. Ingram, Ian Storey, Stefano F. Cappa, Eleonora Catricala, Ajay Halai, Karalyn E. Patterson, Matthew A. Lambon Ralph, James B. Rowe, Peter Garrard

**Affiliations:** 1 Molecular and Clinical Sciences Research Institute, St George’s, University of London, London SW17 0RE, UK; 2 Department of Clinical Neurosciences and Cambridge University Hospitals NHS Trust, University of Cambridge, Cambridge CB2 0SP, UK; 3 Division of Neuroscience and Experimental Psychology, University of Manchester, Manchester M13 9PL, UK; 4 University Institute for Advanced Studies IUSS, Pavia, Italy; 5 IRCCS Mondino Foundation, Pavia, Italy; 6 MRC Cognition and Brain Sciences Unit, University of Cambridge, Cambridge CB2 7EF, UK

**Keywords:** frontotemporal dementia, primary progressive aphasia, random forest classifier

## Abstract

There are few available methods for qualitatively evaluating patients with primary progressive aphasia. Commonly adopted approaches are time-consuming, of limited accuracy or designed to assess different patient populations. This paper introduces a new clinical test—the Mini Linguistic State Examination—which was designed uniquely to enable a clinician to assess and subclassify both classical and mixed presentations of primary progressive aphasia. The adoption of a novel assessment method (error classification) greatly amplifies the clinical information that can be derived from a set of standard linguistic tasks and allows a five-dimensional profile to be defined. Fifty-four patients and 30 matched controls were recruited. Five domains of language competence (motor speech, phonology, semantics, syntax and working memory) were assessed using a sequence of 11 distinct linguistic assays. A random forest classification was used to assess the diagnostic accuracy for predicting primary progressive aphasia subtypes and create a decision tree as a guide to clinical classification. The random forest prediction model was 96% accurate overall (92% for the logopenic variant, 93% for the semantic variant and 98% for the non-fluent variant). The derived decision tree produced a correct classification of 91% of participants whose data were not included in the training set. The Mini Linguistic State Examination is a new cognitive test incorporating a novel and powerful, yet straightforward, approach to scoring. Rigorous assessment of its diagnostic accuracy confirmed excellent matching of primary progressive aphasia syndromes to clinical gold standard diagnoses. Adoption of the Mini Linguistic State Examination by clinicians will have a decisive impact on the consistency and uniformity with which patients can be described clinically. It will also facilitate screening for cohort-based research, including future therapeutic trials, and is suitable for describing, quantifying and monitoring language deficits in other brain disorders.

## Teaser

Patel *et al.* present a new cognitive test for classifying primary progressive aphasia and characterizing language deficits in other brain disorders associated with language impairment. The Mini Linguistic State Examination is brief, accurate and reproducible, and will be useful in profiling language disorders in a variety of clinical settings.

## Graphical Abstract

**Graphical Abstract:**
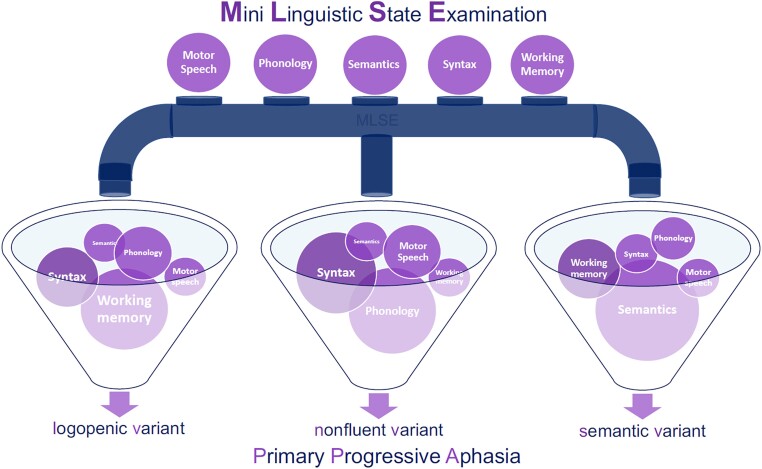

## Introduction

The pathological changes of Alzheimer’s disease and frontotemporal dementia can present with isolated difficulty in language production and/or comprehension—a syndrome referred to as ‘primary progressive aphasia’ (PPA).^1^ A World Federation of Neurology working group defined three distinct subtypes of the phenomenon: the non-fluent variant primary progressive aphasia (nfvPPA) is characterized by effortful and/or agrammatic language production; the semantic variant primary progressive aphasia (svPPA) by anomia and impaired word comprehension and the logopenic variant primary progressive aphasia (lvPPA) by word retrieval and sentence repetition deficits.^2^

The core features distinguishing svPPA, nfvPPA and lvPPA can be reliably detected and quantified using validated test batteries such as the Boston Diagnostic Aphasia Examination^3^ (BDAE) or the Western Aphasia Battery,^4^ though administration and interpretation of such instruments are time-consuming and dependent on specialist expertise that is not widely accessible. Available aphasia scales either provide standardized estimates of severity or were developed specifically to characterize post-stroke aphasia.^5–7^ Formal analysis of connected speech would, unless fully automated, be onerous and operator dependent.^8,9^

In practice, clinical classification is more often based on an informal assessment, though this inevitably leads to inconsistencies and also requires specialist knowledge. Inconsistency and dependence on centralized expertise have impeded wider dissemination of the clinical language assessment skills essential to clear communication in the clinical domain. There is, therefore, a pressing need for a clinical instrument that enables the description and diagnosis of aphasias in a harmonized, efficient and quantifiable fashion. The need will be further amplified by the requirement to screen for PPA subtypes when disease-modifying therapies come to be developed and tested.

We developed the Mini Linguistic State Examination (MLSE) as a method of profiling PPA consistently, quantitatively and reproducibly. We designed the MLSE to be brief, usable by non-specialists after minimal training, and not only sensitive to the three archetypal syndromes but also able to detect and define atypical symptom clusters. Finally, and in a departure from conventional clinical scoring methods based on response accuracy, we proposed that recording the rates at which different types of errors were made by a participant would yield a high level of discrimination.

By way of a preliminary study of the construct validity of the MLSE, the present paper describes the test and reports the profiles obtained in a cohort of patients with predominantly mild PPA, recruited through specialist cognitive neurology services at three centres in the UK. The paper reports statistics relating to the validity, reproducibility, accuracy and ease of administration of the MLSE and the output of a machine-learning-derived decision tree to classify the PPA subtypes using data obtained from administering the test.

## Participants, materials and methods

### Participants

A total of 61 patients with one of the three canonical variants of PPA (25 lvPPA, 20 nfvPPA, 16 svPPA) were recruited through cognitive neurology clinics at St George’s Hospital, London (*n* = 26), Addenbrooke’s Hospital, Cambridge (*n* = 27) and Manchester Royal Infirmary and its associated clinical providers (*n* = 8). Diagnosis was based on the WFN working group criteria,^2^ including brain imaging, neuropsychological assessment and clinical review by multidisciplinary teams. Three patients declared a native language other than English but were highly fluent, had been communicating in English since childhood and predominantly or exclusively used English in day-to-day life. Three patients and four controls subjects were left handed. Seven patients were excluded due to the advanced stage of their condition (4 × lvPPA, 3 × nfvPPA) leaving 54 PPA patients in the final analysis. Patients with PPA who did not meet diagnostic criteria for one of the three canonical variants (i.e. those with a mixed phenotype) were not recruited. The number of patients with a mixed phenotype was not recorded.

Thirty healthy volunteers were recruited through the National Institute for Health Research ‘Join Dementia Research’ registers in London and Cambridge and invitations to patients’ relatives. Controls had no history of significant neurological, psychological, speech and language or learning deficits. All were native speakers of English with normal or corrected-to-normal hearing and vision.

Written informed consent was provided by all participants. The study protocol was reviewed and approved by the London (Chelsea) Research Ethics Committee [Ref. 16/LO/1735]. The study was sponsored by St George’s, University of London, the University of Cambridge and the University of Manchester.

### Experimental design

Participants underwent baseline assessments using the Addenbrooke’s Cognitive examination, version 3 (ACE-III) and the short form of the BDAE.^10,11^ If a participant had completed the ACE-III within a month prior to performing the MLSE, the ACE-III version B was administered.

### The Mini Linguistic State Examination

The MLSE, together with the administration and scoring guide, can be downloaded from Supplementary material and can be freely used for non-commercial purposes. The test consists of 11 subtests, each of which makes a different combination of demands on the components of language competence affected by PPA.^2^ As there are few individual tests of language production or comprehension that are selectively sensitive to any component of linguistic competence in isolation, the MLSE captures the nature of a patient’s language impairment on the basis of the number and nature of errors made during the response. Five types of error are considered, reflecting dysfunction of: (i) the motoric aspects of speech; (ii) semantic knowledge; (iii) knowledge of phonology; (iv) knowledge of syntax and (v) auditory-verbal working memory. The 11 subtests are: (1) picture naming (six items); (2) syllable and multisyllable repetition (three items); (3) word repetition combined with single-word comprehension (‘Repeat and point’) (three items); (4) non-word repetition (three items); (5) non-verbal semantic association (four items); (6) sentence comprehension (verbal) (four items); (7) sentence comprehension (pictorial) (four items); (8) word and non-word reading (10 items); (9) sentence repetition (four items); (10) writing (one item) and (11) picture description (one item).

The method generates a profile score that reflects performance within five domains of linguistic competence, as well as an overall score reflecting the severity of the disorder.

General definitions of the five error types are provided in Table 1, along with the subtests on which it is possible to commit each type of error. Additionally, because the circumstances under which errors occur differ across tasks (e.g. between written and spoken tasks or between those requiring verbal versus non-verbal responses), definitions specific to each subtest are also specified, with examples, in the administration and scoring guide.

**Table 1:** General definitions of the five types of errors that are recorded during administration of the MLSE

	Definition	Notes	Subtests in which errors can be made (max errors in each)
Motor speech error	A response that is slurred, stuttered or contorted and which the examiner would find difficult to repeat or transcribe	Motor speech errors arise only during tasks requiring speech production.A motor speech error should be noted and scored, even when self-corrected. The errors are not confined to speech dyspraxia	Naming (6)Syllable repetition (3)Repeat and point (3)Non-word repetition (3)Reading (10)Sentence repetition (4)Picture description (1)
Phonological error	A response that contains incorrect but word-like components and which could easily be repeated or written down	Phonological errors arise only during tasks requiring speech production.Any phonological error should be noted and scored, even when self-corrected	Naming (6)Syllable repetition (3)Repeat and point (3)Non-word repetition (3)Reading (10)Sentence repetition (4)Picture description (1)
Semantic error	A semantic error is noted when a participant’s response suggests a deficit at the level of conceptual knowledge and/or word meaning	Semantic errors can arise during both production (e.g. naming) and comprehension (e.g. picture association) tasks. Context-specific guidance is provided for each subtask	Naming (6)Repeat and point (3)Semantic association (4)Reading (5)Picture description (2)
Syntactic error	A syntactic error occurs when a participant demonstrates difficulty understanding or producing grammatically correct sentences	Context-specific guidance is provided for each subtask	Sentence comprehension (8)Writing (1)Picture description (1)
Working memory error	Working memory errors are recorded when a participant is unable to repeat sentences correctly. The shorter the incorrectly repeated sentence, the higher the error score	Working memory errors are scored only during the sentence repetition task	Sentence repetition (10)

### Scoring the MLSE

A participant’s profile was determined by subtracting the number of errors of each type from the number of opportunities to make such an error. If a participant made no errors, the test would yield a profile score of 30/30 for motor speech, 30/30 for phonology, 20/20 for semantics, 10/10 for syntax, 10/10 for working memory and an overall score of 100/100. Multiple error types can be associated with a single response: for instance, in the naming task, if a participant were to produce a semantic substitution that contained a phonological error, both a semantic and a phonological error would be recorded (see Supplementary Tables 1 and 2).

Some patients with advanced PPA were unable to make any response, even with encouragement from the tester. When this occurs, the test item is associated with a ‘no-response’ error, which is equivalent to the sum of all possible domain error scores for that item. The seven PPA patients excluded from the analysis were those whose scores included ‘no-response’ errors. Example scoring of the ‘no-response’ errors can be found in Supplementary Fig. 1.

Testing was performed in a quiet environment and video and/or audio recorded to enable offline scoring and between-rater agreement measures. Recordings of 30 patient evaluations were used to perform independent parallel evaluations by three different raters (one from each site) blinded to the syndromic diagnosis.

### Statistical analysis

Data were analysed using IBM SPSS (version 25.0). Convergent validity was measured using Cronbach’s alpha^12^ and through correlation of standardized scores obtained in subtasks of the MLSE with relevant subsections of established measures (BDAE and ACE-III/R). Specifically, correlations between the following pairs of tests (components of the BDAE and MLSE, respectively) were conducted: repetition of single words and the repetition component of the repeat and point subtest; auditory comprehension and the pointing component of the repeat and point subtest; repetition of sentences and the sentence repetition subtest; the Boston naming test and the naming subtest; oral reading and the reading subtest. The sentence repetition subtest was compared with working memory components of the ACE-III/R (namely, the sum of the scores achieved on repetition of word-lists, sentences and the name and address).

Inter-rater reliability was obtained using a random intraclass correlation (ICC) model based on absolute agreement. Demographic characteristics and all test-derived scores were compared across groups using Welch’s ANOVA due to unequal variances and sample size per group (giving the asymptotically *F* distributed score), and *post hoc* pairwise comparisons with the Bonferroni correction. Socio-demographic variables were compared using parametric or non-parametric tests depending on Levene’s test for equality of variance. Receiver-operating characteristic (ROC) curves were plotted to assess the differential diagnostic efficiency of different features. Discriminant function analysis was conducted to demonstrate the classification accuracy of the three PPA subtypes.

### Machine-learning classification

A random forest (RF) classifier was trained and tested using MATLAB (2019a, version 25.0). The RF classification method has been applied extensively to medical data because of its accuracy, robustness to noisy datasets and relative immunity to overfitting.^13,14^ The full sample was split randomly (weighted by the numbers in each diagnostic group) into a training (60%, *n* = 50) and out-of-sample test set (40%, *n* = 34). The training test was used for training the model using 5-fold leave-one-out cross-validation. The trained model was then evaluated against the out-of-sample data (see Supplementary Table 10).

The RF consisted of 100 decision trees, a number determined through a grid search in which a range of forests are grown containing *n* trees, where *n* begins at 10 and increments to a maximum of 1000. The number of predictors to sample was set equal to the square root of the total number of features.^15^ Sensitivity, specificity, *F*1-score, precision, recall and balanced classification accuracy were used as evaluation metrics of average fold performance for each experiment, as well as final model testing, after manual selection of domain combinations with high-balanced accuracy. The final tree structure is identified by testing each decision tree within the forest and calculating the average and variance between class accuracies of the out-of-sample testing data. The final model was also used to create a clinical decision tree to guide the manual classification of new test data.

### Data management

Study data were collected and managed using the Research Electronic Data Capture (REDCap) tool hosted at St George’s, University of London and the University of Cambridge.^16^

### Data availability

Anonymized data are available on reasonable request for academic purposes.

## Results

### Participant characteristics

Group characteristics are displayed in Table 2. Age, years of education and time since diagnosis were similar across the whole patient and control groups (*P* > 0.05). Comparing across patient groups, svPPA patients tended to be younger [median (interquartile range, IQR) age in years = 65 (63–70)] than both the lvPPA [73 (67–79), *P* = 0.01] and nfvPPA patients [71 (66–73), *P* = 0.09]. Symptom duration was longer for svPPA [mean (SD): 5.8 (4)] than lvPPA [2.4 (2), *P* = 0.009], but not nfvPPA [3.1 (2), *P* = 0.409]. Cognitive characteristics revealed by BDAE and ACE scores per PPA subtype are presented in Table 2.

**Table 2:** Demographics and general cognitive characteristics for each PPA subtype and healthy controls

	lvPPA	nfvPPA	svPPA	Controls
No. of participants	21	17	16	30
Age, mean (SD)	73 (67–79)	71 (66–73)	65 (63–70)	68 (65–70)
Sex, male:female	15:6	6:11	8:8	18:12
Handedness, right:left	19:1	15:2	17:0	27:3
Education (years), mean (SD)	19 (3)	17 (2)	19 (2)	21 (3)
Time since diagnosis (years), mean (SD)	1.2 (1)	2 (1.7)	2.4 (2)	—
Language symptom onset (years), mean (SD)	2.4 (2)	3.1 (2)	5.8 (4)	—
BDAE sub-scores, mean (SD)				
Repetition of single words (/5)	4 (0.6)	4 (1)	4 (0.8)	5 (0)
Auditory comprehension (/16)	15 (2)	14 (4)	11 (3)	16 (0.2)
Picture–word matching (/4)	3 (1)	3 (1)	2 (1)	4 (0.3)
Repetition of sentences (/2)	1 (0.6)	1 (0.7)	2 (0.6)	2 (0)
Boston naming test (/15)	8 (4)	9 (5)	3 (3)	14 (0.4)
Oral reading (/15)	14 (2)	12 (5)	13 (3)	15 (0)
ACE-III/R sub-scores mean (SD)				
Attention (/18)	12 (3)	13 (5)	15 (2)	18 (0.6)
Memory (/26)	9 (7)	14 (8)	9 (5)	25 (0.7)
Fluency (/14)	4 (3)	3 (3)	4 (2)	13 (0.3)
Language (/26)	18 (5)	18 (6)	11 (3)	26 (0.4)
Visuospatial (/16)	12 (2)	12 (5)	15 (1)	16 (0)

lvPPA, logopenic variant PPA; nfvPPA, non-fluent variant PPA; svPPA, semantic variant PPA; BDAE, Boston diagnostic aphasia examination; ACE, Addenbrooke’s cognitive examination; SD, standard deviation.

### Test characteristics

Administration of the MLSE took an average (SD, median, range) of 19 (3, 19, 13–24) min, with lvPPA taking longest at 20 (3, 20, 14–22) min, followed by svPPA at 19 (2, 19, 13–24) and nfvPPA 18 (2, 18, 14–21) min.

A two-way mixed-effects model (people effects are random and measures effects are fixed) showed scoring decisions made by the three independent raters to be highly consistent, with an ICC index of 0.95 (*P* < 0.0001).

The reliability of the MLSE against the BDAE and ACE for all participants resulted in a Cronbach’s alpha score of 0.908. Convergent validity produced correlations ranging from 0.603 to 0.669. Correlations between test pairs were: 0.665 for single-word repetition; 0.669 for auditory comprehension; 0.613 for sentence repetition; 0.663 for picture naming; 0.603 for word reading and 0.632 for working memory (*P* < 0.001 for all correlations).

### Language profiles

**Figure 1:**
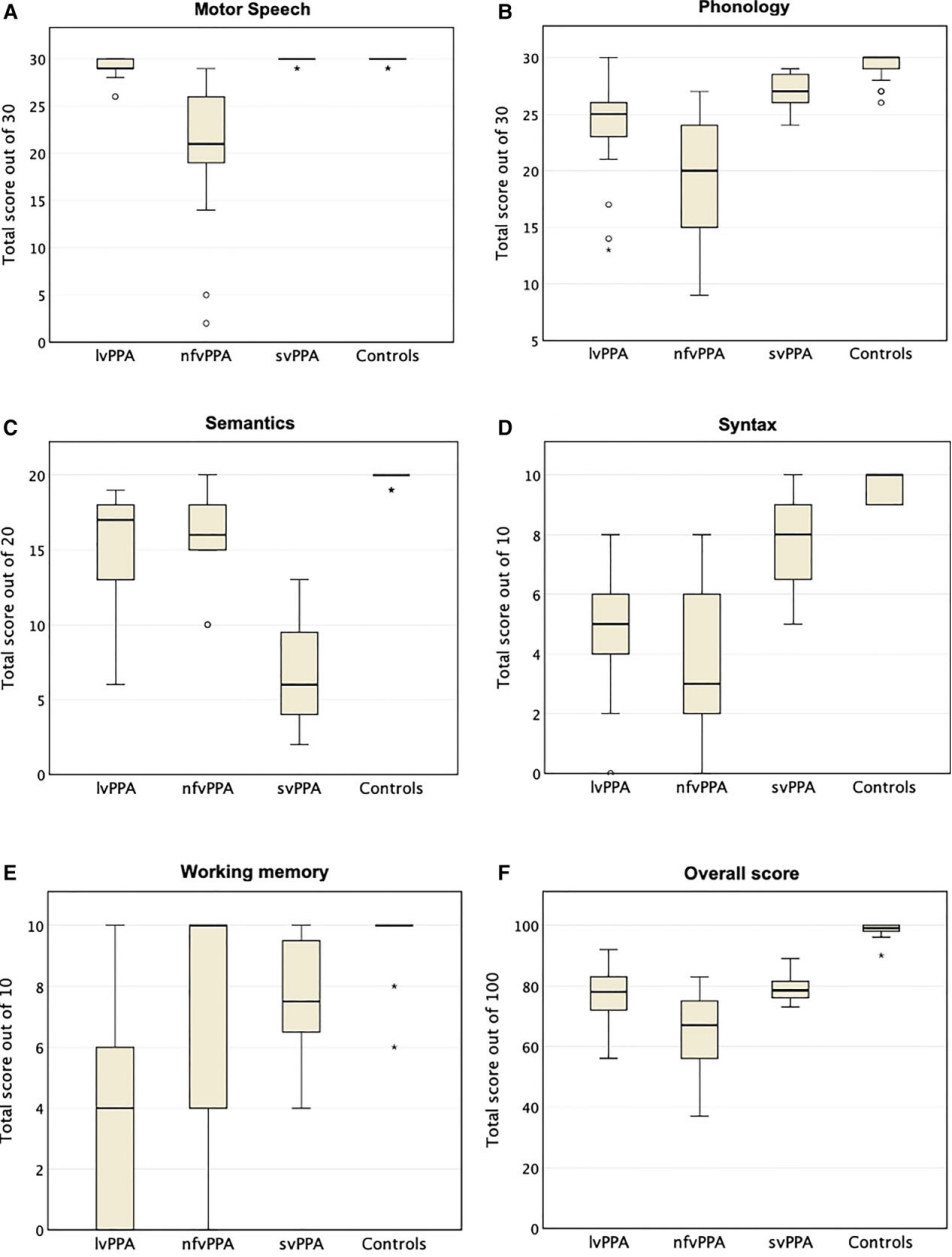
**MLSE domain scores (A–E) and total score (F) grouped by diagnosis.** The boxes represent IQRs, horizontal lines the medians and error bars the minimum and maximum values excluding outliers. The latter are represented by the symbols ‘circle’ (values which are between 1.5 and 3.0 times the IQR below the first quartile or above the third) and ‘asterisk’ (values which are >3.0 times the IQR below the first quartile or above the third).

Scores grouped by diagnosis in each of the five linguistic domains are presented in Fig. 1 along with group medians and IQRs for individual domains and overall MLSE score. The average total MLSE scores [median (IQR)] were: svPPA = 79 (76–82), lvPPA = 78 (71–84) and nfvPPA 67 (55–76): *F*(3,80) = 137.11 (*P <* 0.001). These overall scores were higher in svPPA and lvPPA compared to nfvPPA (*P* = 0.002 and 0.019, respectively).

**Figure 2:**
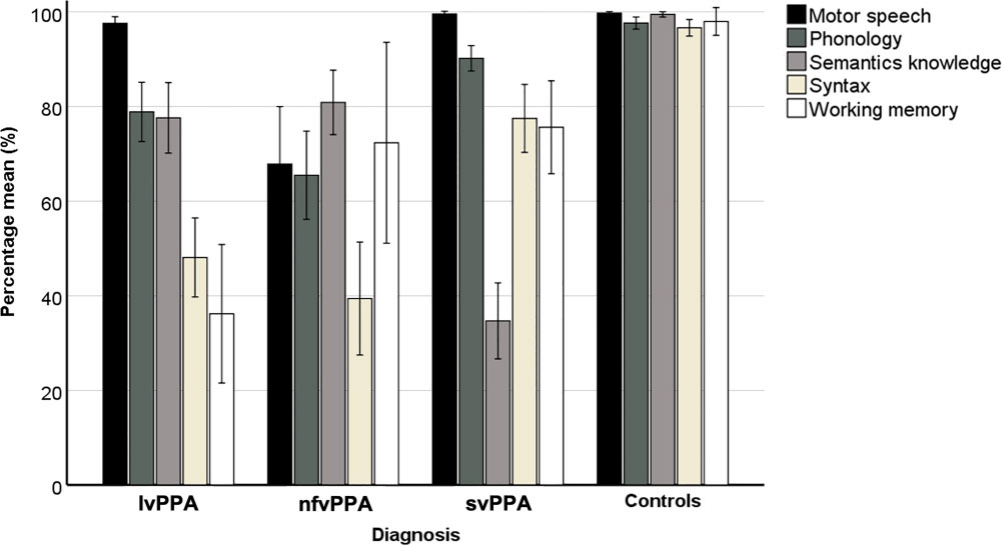
**MLSE results.** Mean percentage scores with error bars showing standard deviations in five linguistic domains grouped by PPA subtype and healthy controls. lvPPA, logopenic variant PPA; nfvPPA, non-fluent variant PPA; svPPA, semantic variant PPA.

The distribution of individual domain scores (expressed as percentages of maximum scores) is presented in Fig. 2. There were significant group differences associated with all domains:

- *Motor speech F*(3,80) = 11.72 (*P <* 0.001): the nfvPPA group [percentage mean (SD), 67 (25)] scored significantly lower than both lvPPA [97 (3)] and svPPA [99 (1)], (both *P* < 0.001), and there was a marginal difference in motor speech scores between lvPPA and svPPA (*P* = 0.066).
- *Phonology F*(3,80) = 30.83 (*P <* 0.001): the nfvPPA group [65 (19)] scored lower than lvPPA [78 (14)] but this was not statistically significant (*P* > 0.05). However, both the nfvPPA group and the lvPPA group scored significantly lower than svPPA [90 (5)] (*P* < 0.01 for both contrasts).
- *Semantic knowledge F*(3,80) = 102.05 (*P <* 0.001): svPPA patients [34 (16)] scored significantly lower than lvPPA [77 (17)] and nfvPPA patients [80 (14)] (*P* < 0.001 for both contrasts). There was no significant difference in semantic knowledge scores between lvPPA and nfvPPA patients (*P* > 0.05).
- *Syntax F*(3,80) = 74.11 (*P <* 0.001): scores were significantly lower in lvPPA patients [48 (19)] and nfvPPA patients [39 (24)] than in the svPPA group [76 (14)] (both *P* < 0.001). There was no significant difference in syntax domain scores between nfvPPA and lvPPA patients (*P* > 0.05).
- *Working memory F*(3,80) = 28.06 (*P <* 0.001): scores were lowest in the lvPPA group [36 (33)] and statistically different from both nfvPPA [72 (43)] and svPPA [75 (19)] (*P* < 0.05 and <0.001, respectively). There was no significant difference in working memory between nfvPPA and svPPA (*P* > 0.05).

### Diagnostic accuracy

**Figure 3:**
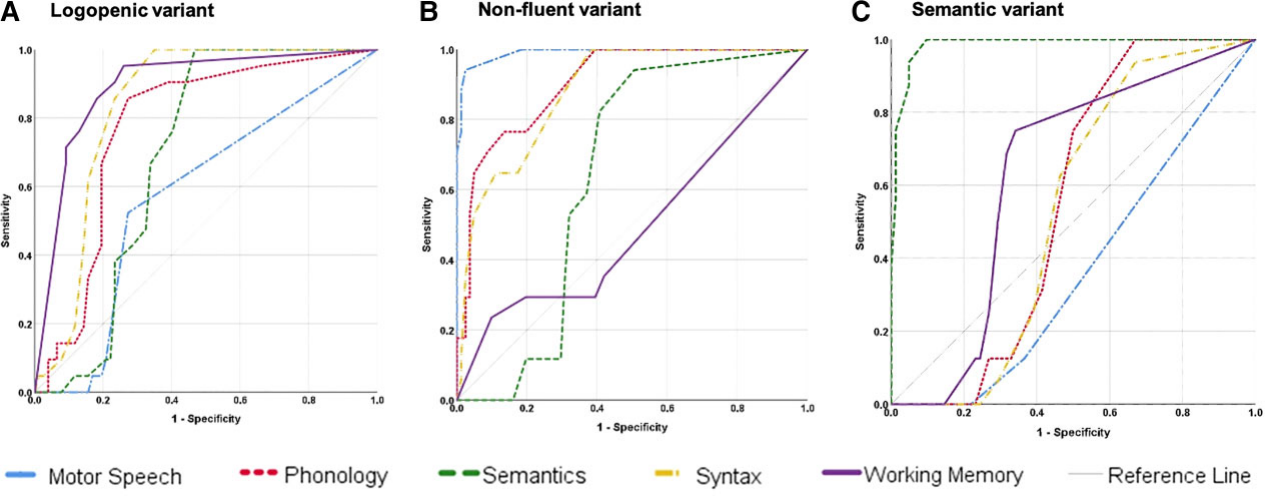
**Domain accuracies.** Independent ROC curves demonstrating the accuracy of all five linguistic domains for each PPA subtype.

ROC analysis (see Fig. 3A–C) revealed that phonology [area under the curve (AUC) = 0.77], syntax (AUC = 0.84) and working memory (AUC = 0.89) were the best parameters for the diagnosis of lvPPA (all *P*<0.001). For the diagnosis of nfvPPA, motor speech (AUC = 0.99), phonology (AUC = 0.90) and syntax (AUC = 0.88) were all good parameters (all *P*<0.001), whilst semantic knowledge (AUC = 0.99) was the best parameter for the diagnosis of svPPA (*P* < 0.001).

### Machine-learning classification

To further explore the diagnostic accuracy of the MLSE, a robust machine-learning method for feature selection and RF tuning was conducted based on the five linguistic domains. The predictive capacity of the resulting model was excellent, with an overall accuracy of 0.96. All controls were correctly classified. Diagnostic accuracies for each of the three syndromes (Table 3) were 0.92 for lvPPA (89% correctly classified; one patient misclassified as nfvPPA; one false positive from the svPPA group); 0.93 for svPPA (86% correctly classified; one patient misclassified as lvPPA) and 0.98 for nfvPPA (100% correct classification and one false positive from the lvPPA group).

**Table 3:** Confusion matrix for predicting PPA diagnosis for 34 participants using random forests classification.

		Predicted diagnosis				
		lvPPA, *n* (%)	nfvPPA, *n* (%)	svPPA, *n* (%)	Controls, *n* (%)	Accuracy
Actual diagnosis	lvPPA, *n* (%)	**8** (**89)**	1 (11)	0 (0)	0 (0)	0.924
	nfvPPA, *n* (%)	0 (0)	**7 (100)**	0 (0)	0 (0)	0.981
	svPPA, *n* (%)	1 (14)	0	**6** (**86)**	0 (0)	0.928
	Controls, *n* (%)	0 (0)	0 (0)	0 (0)	**11** (**100)**	1.000

The overall balanced accuracy of the model was 0.958. True positives in bold type. lvPPA, logopenic variant PPA; nfvPPA, non-fluent variant PPA; svPPA, semantic variant PPA.

A final set of feature rankings for each domain was selected from the results of the training (*k*-fold) procedure and used in the evaluation of the unseen, out-of-sample set. Balanced accuracy varied as the number of domains reduced. The svPPA and control models showed the highest balanced accuracy when using all five domains. The nfvPPA model showed the highest balanced accuracy when using four domains (motor speech, phonology, syntax and working memory: 0.943). The lvPPA model achieved the highest balanced accuracy with three domains (syntax, working memory and motor speech: 0.944). A detailed description of the analysis can be found in Supplementary Fig. 4.

**Figure 4:**
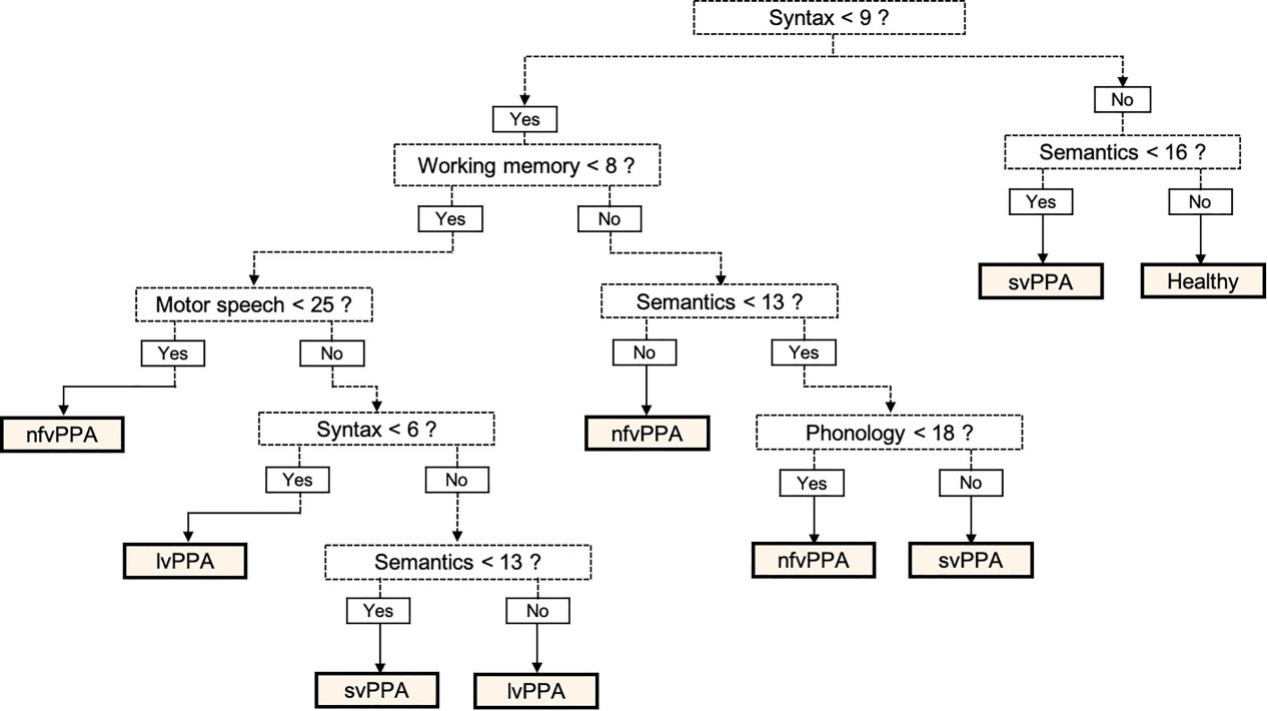
**MLSE diagnostic decision tree.** On the scores of the five linguistic domains to classify PPA subtypes from the out-of-sample data, this decision tree yielded correct classifications of 91% (31/34 participants—9 lvPPA, 7 svPPA, 7, nfvPPA, 11 controls). lvPPA, logopenic variant PPA; nfvPPA, non-fluent variant PPA; svPPA, semantic variant PPA.

Whilst the RF classifier is robust and accurate, it does not produce readily interpretable diagnostic rules. A decision tree structure was therefore selected from amongst the RFs as a guide to manual classification of PPA subtypes from MLSE scores. The tree (Fig. 4) was selected for its accuracy, simplicity and the fact that a diagnosis was made using all five linguistic domains. This decision tree correctly classified 91% (31/34) of the patients and controls whose data were not included in the training set. Two misclassifications were in the lvPPA group (lvPPA2 and lvPPA20 misclassified as nfvPPA). Both of these lvPPA patients scored highly in the working memory and phonology domains. One svPPA patient, who showed deficits in the syntax and working memory domains, was misclassified as lvPPA.

## Discussion

This paper reports the motivation, assumptions, structure and diagnostic properties of a clinical instrument that can be used for detection, diagnosis and classification of patients with the classical syndromes of PPA. The MLSE was motivated by the need for a brief, reliable and reproducible measure of language competence that is differentially sensitive to the classic PPA subsyndromes and enables a clinician quantitatively to assess the components of linguistic competence whose dysfunction characterizes each of these variants.

Competence in the domains of motor speech, phonology, semantics, syntax and auditory-verbal working memory, which are differentially impaired across the PPA variants,^17–23^ is quantified in the MLSE in terms of the numbers of errors deriving from each domain that a patient makes during a sequence of 11 simple linguistic assays. The error-based approach to scoring maximizes the clinical information available from any single test condition without prolonging the duration of administration.

Whilst assigning the origin of an error to a specific domain is, in principle, subject to disagreements between individual assessors, we found that simple, rule-based guidance led to a high level of consistency amongst three junior researchers (two postdoctoral and one predoctoral), all of whom had previous experience in cognitive assessment, but none specifically in language. The validity of error-based measurement is also supported by the fact that performance scores on subtests of more established assessment instruments (BDAE and ACE-III) showed a good correlation with those derived from the error-based method.

The MLSE was able to distinguish patients with mild PPA from age-matched, control participants with 100% accuracy and based on the distributions of error types across the three variants, an RF classifier assigned the correct diagnosis to 21 of 23 patients (91%) from an out-of-sample group. svPPA can be a relatively straightforward diagnosis for an experienced clinician, and the MLSE reproduced the characteristic, and more or less isolated, impairment of semantic knowledge on which this diagnosis is largely based. More challenging has been the distinction between nfvPPA and lvPPA,^24^ as phonology is impaired in both syndromes. That the MLSE can distinguish effectively between these two syndromes is largely due to the fact that motor speech and working memory are also quantified, contributing to a 0.98 accurate classification of nfvPPA, with only one lvPPA placed erroneously into this group.

With its proven ability to reproduce an expert clinical diagnosis, the MLSE can provide clinicians who do not have specialist knowledge of language and/or cognitive disorders with the means to make accurate, consensus-based classifications as part of a routine outpatient assessment. An equally important contribution to neurological practice, however, is the detailed and consistent descriptive vocabulary for characterizing language disorders of any aetiology.^25^ Whilst the patients reported here were included because their cognitive disorder was clearly an accepted variant of PPA, progressive language disorders that cannot be assigned to any of these categories (‘mixed PPA’) can also be clearly described and new syndromic subtypes delineated.^26,27^ This property of the MLSE will also aid the clinical assessment of other conditions in which compromised language accompanies movement disorders,^28^ generalized dementia^29^ or behavioural change.^30,31^ A well-documented phenomenon is a presentation of nfvPPA and the later development of the motor features of corticobasal syndrome.^32^ A related prodromal phase has been described for progressive supranuclear palsy.^33–35^ The development of frontal features of disinhibition and/or obsessionality following presentation with ‘pure’ svPPA is also a common clinical sequence.^36,37^

Two patients from the current cohort illustrate that the overlap between PPA and Alzheimer’s disease is more complex than the well-known association with the logopenic variant.^29^ Prominent anomia, fluent but empty speech and impaired semantic knowledge supported an expert clinical diagnosis of svPPA in patients svPPA2 and svPPA3, yet their MLSE profiles revealed, in addition, a low working memory score that was atypical for the group. Biomarkers of Alzheimer’s disease pathology were later identified in the CSF of both these patients.

We have shown how a machine-learning algorithm can learn patterns in data across the five linguistic domains and that the features on which this learning was based coincided with *a priori* definitions of the syndromes.^2,19,20^ An advantage of the RF classifier lies in the assessment of data containing irregular samples or missing data points. It can outperform support vector machines and linear mixed-effects methods and is thus an effective choice for this type of classification challenge.^38^ RF classification was thus shown to be a robust statistical method to demonstrate classification accuracy, though it does not provide easily applicable diagnostic rules. As an aid to clinicians, therefore, a component tree was selected as a simple decision structure for the manual classification of individual cases. Improved accuracy could be achieved by making the full model available in script format to allow optimal classification to be produced for any new combination of domain scores. We intend to make this functionality available in the future.

Further data collection and analyses are also in progress to determine: (i) whether the MLSE can be incorporated into real-world clinical or neuropsychological consultations with equivalent degrees of accuracy and consistency of error assignment (within as well as between individuals) by non-specialist assessors working with the existing error definitions, which—particularly in respect of the distinction between phonological and motor speech errors—are relatively unsophisticated; (ii) whether the MLSE will classify mixed/atypical cases (as determined by an expert clinician) as separate from the canonical diagnostic groupings, or misclassifies such cases as belonging to one of the canonical groups—an issue that can only be resolved by collecting a data set of the MLSE scores of patients with mixed PPA; (iii) whether and to what extent a patient’s profile and/or total score on the MLSE are sensitive to progression of the degenerative process; (iv) whether the patterns of domain competence show the expected spatial correlations with regional grey-matter atrophy on MR imaging and (v) whether its diagnostic accuracy is generalizable to other languages after differential item familiarity, language-dependent vulnerability of different linguistic domains^39^ and the nature of the correspondence between written representations and phonological forms are taken into account.^40^ Versions of the MLSE for Italian and Spanish-speaking populations have already been developed and formal comparisons of the performance of the instrument across these languages are in progress. We encourage the development of versions in other languages, including those outside the Indo-European family. In the meantime, the test and methodology are freely available under a Creative Commons Licence for the purposes of non-commercial research.

## Supplementary Material

fcab299_Supplementary_Data

## Data sharing

All data collected for this study (i.e. deidentified participant data and a data dictionary) will be made available under a signed data access agreement, after the online publication date, in response to all reasonable requests from academic researchers emailed to the corresponding author.

## Abbreviations

ACE-III =: Addenbrooke’s Cognitive Examination, version 3
AUC =: area under the curve
BDAE =: Boston Diagnostic Aphasia Examination
ICC =: intraclass correlation
lvPPA =: logopenic variant primary progressive aphasia
nfvPPA =: non-fluent variant primary progressive aphasia
ROC =: receiver-operating characteristic
RF =: random forest
svPPA =: semantic variant primary progressive aphasia.
