# Supplementary material for: A ‘Mini Linguistic State Examination’ to classify primary progressive aphasia
Source: Brain Commun. 2021 Dec 21;4(2):fcab299. doi: 10.1093/braincomms/fcab299 (PMC8914496; doi:10.1093/braincomms/fcab299)
Supplement: fcab299_Supplementary_Data [file fcab299_supplementary_data.docx]

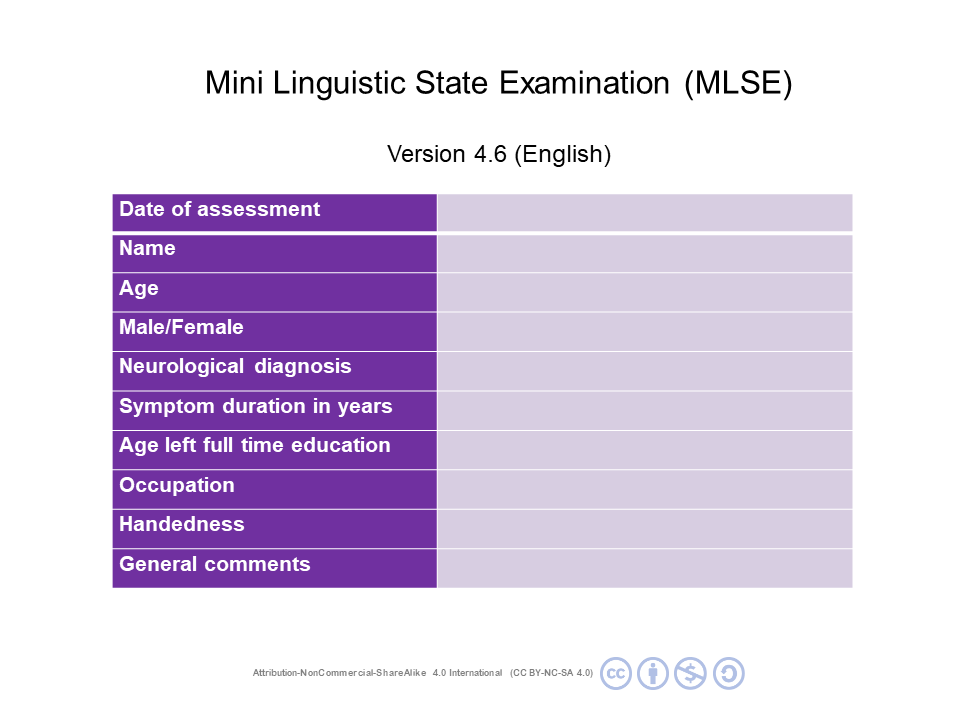


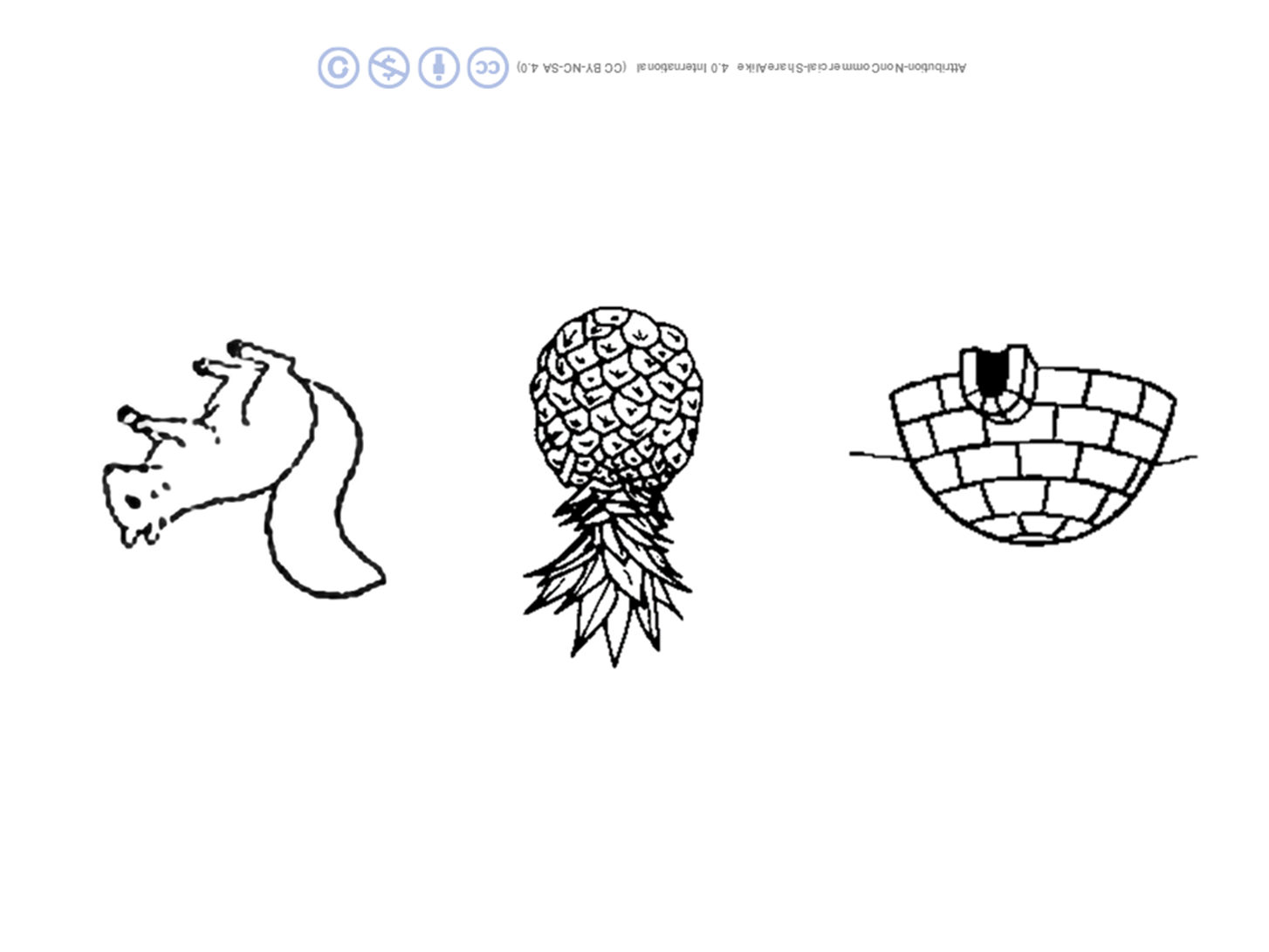


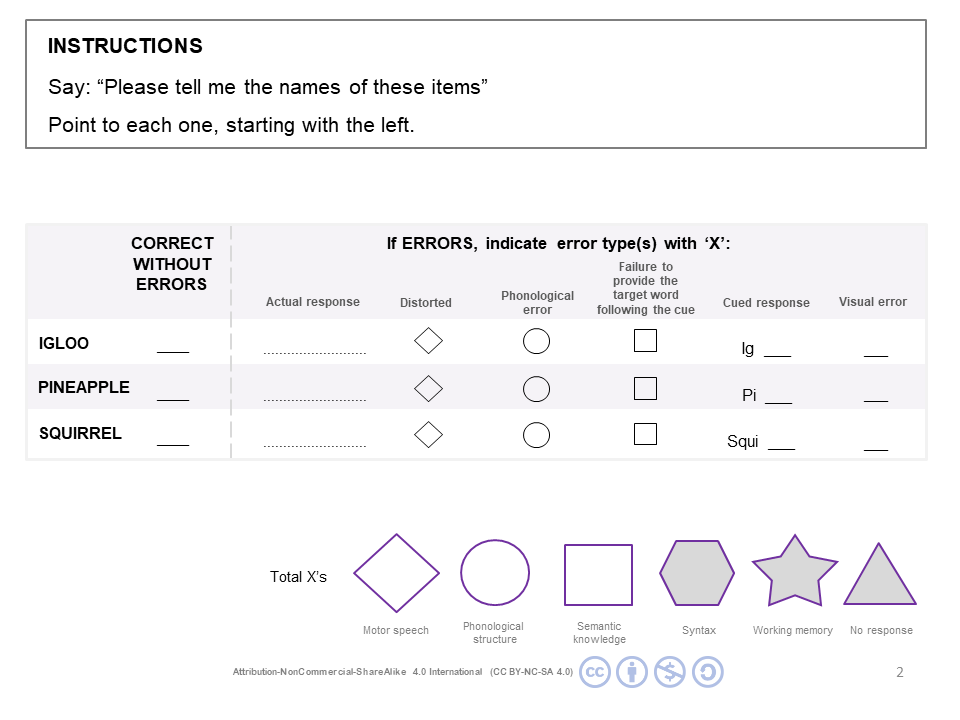


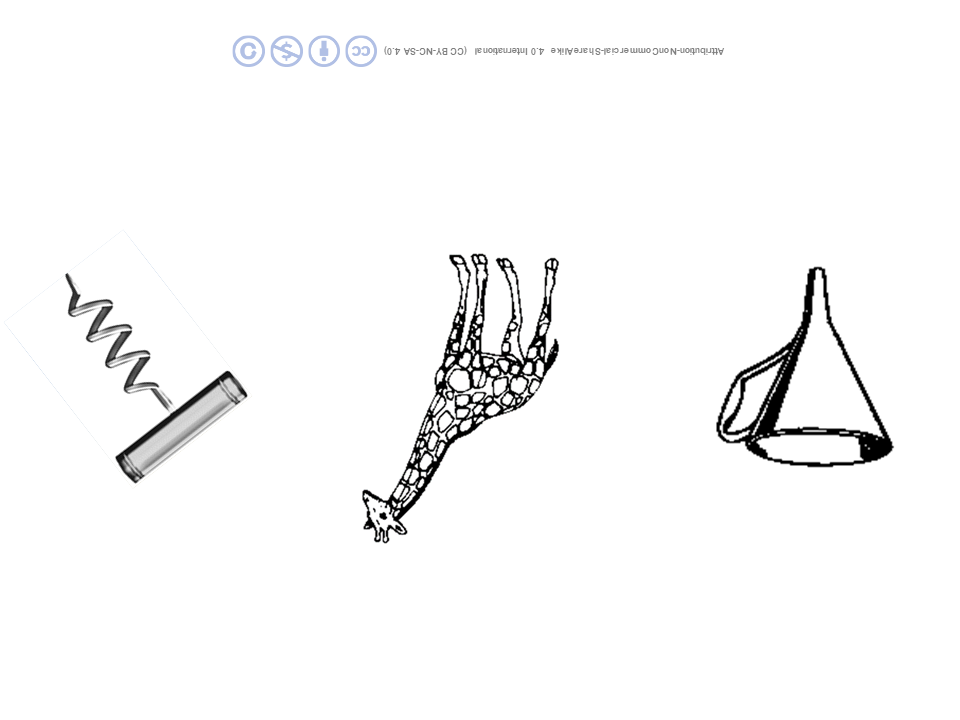


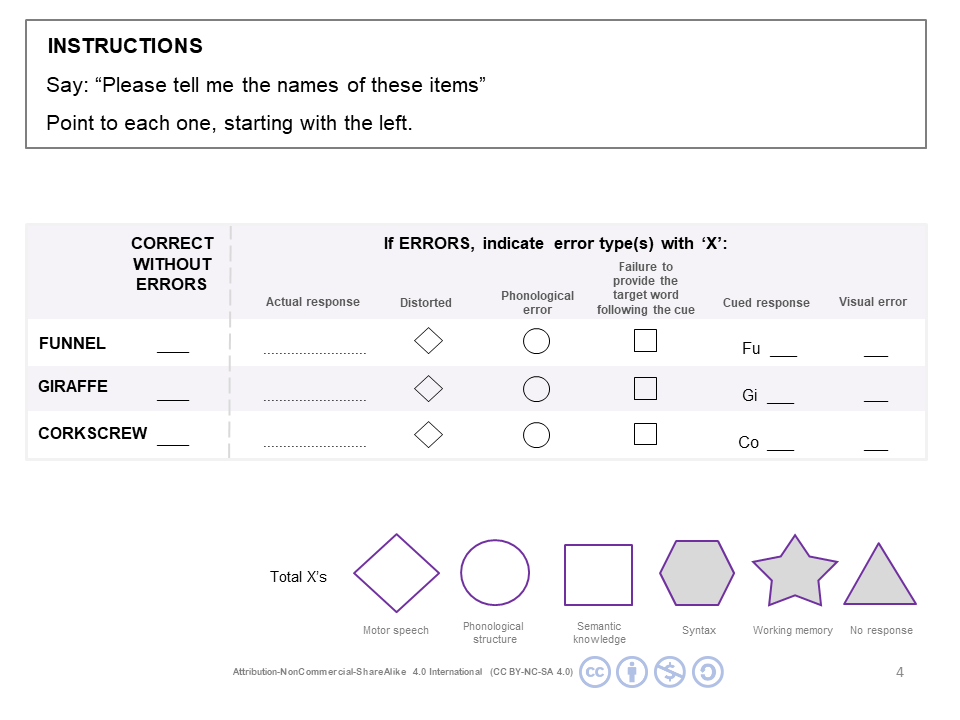


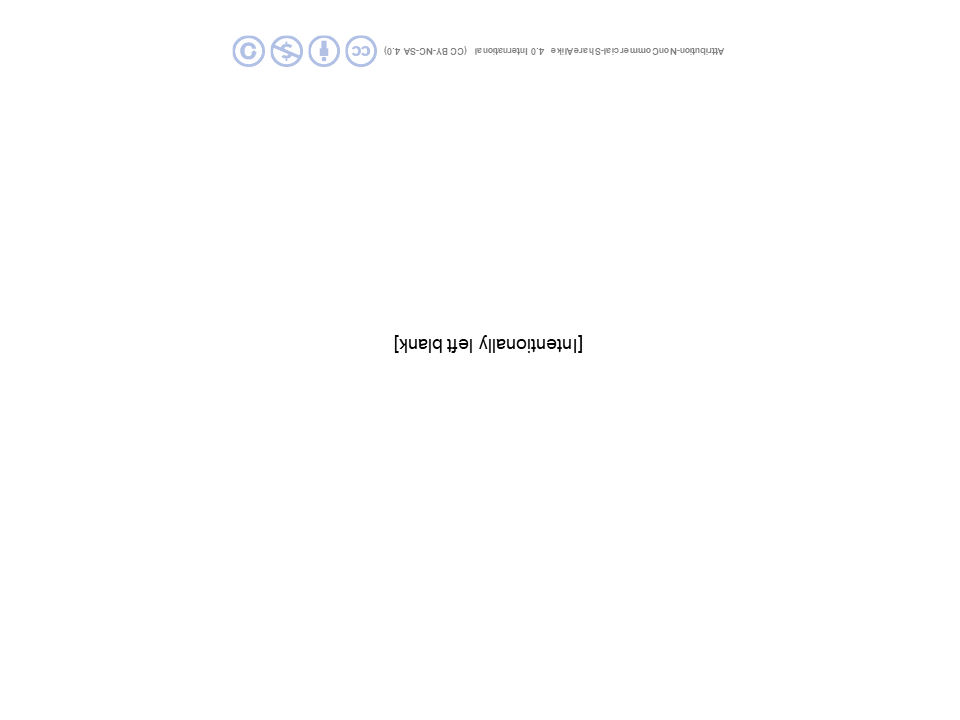


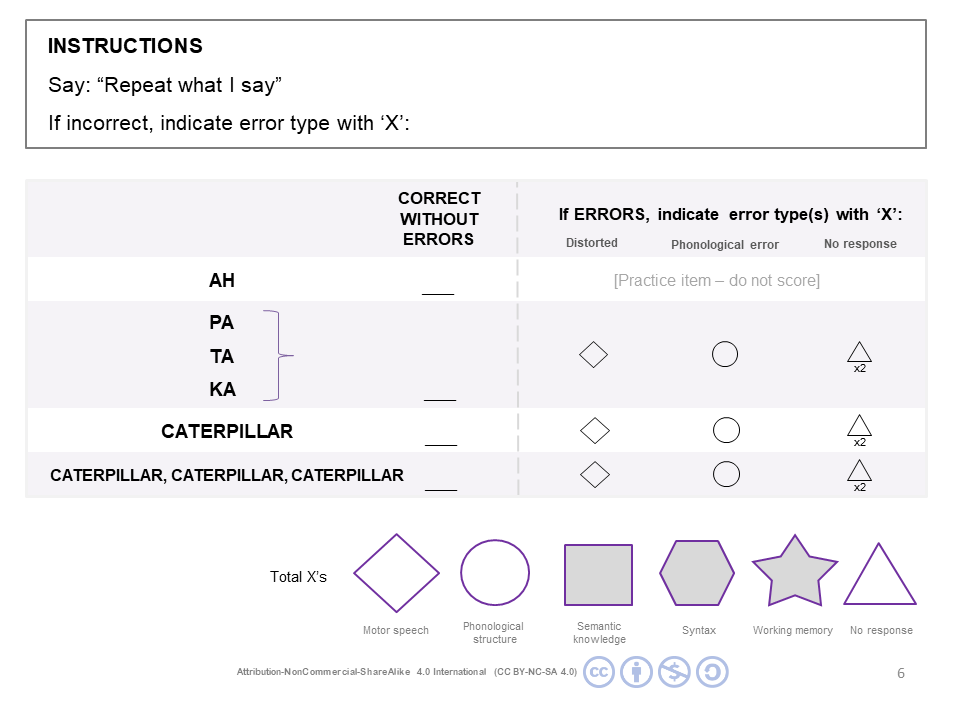


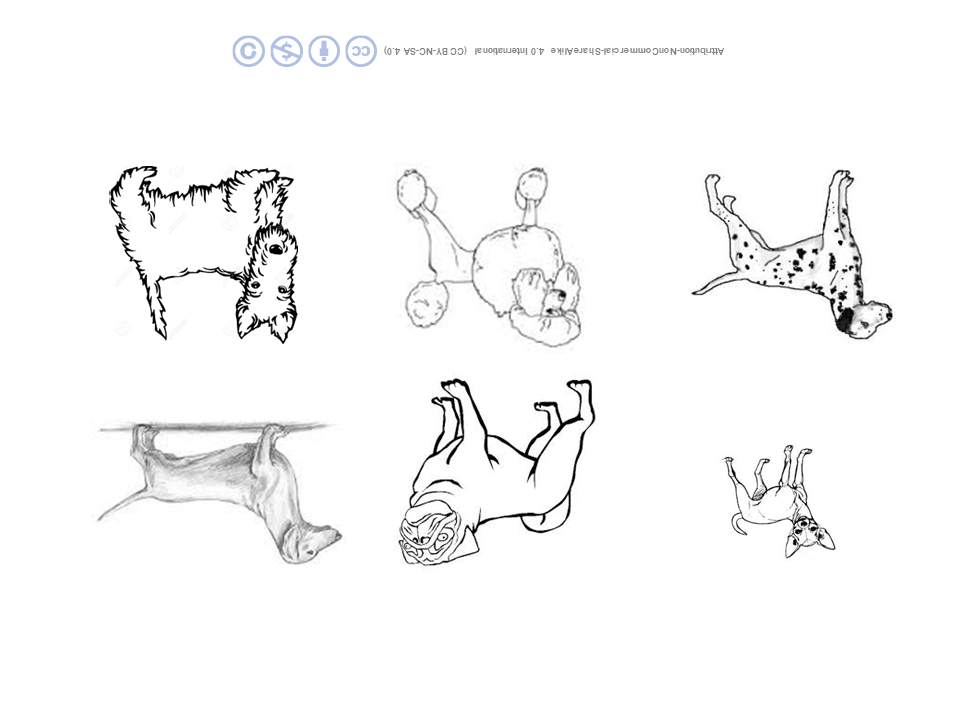


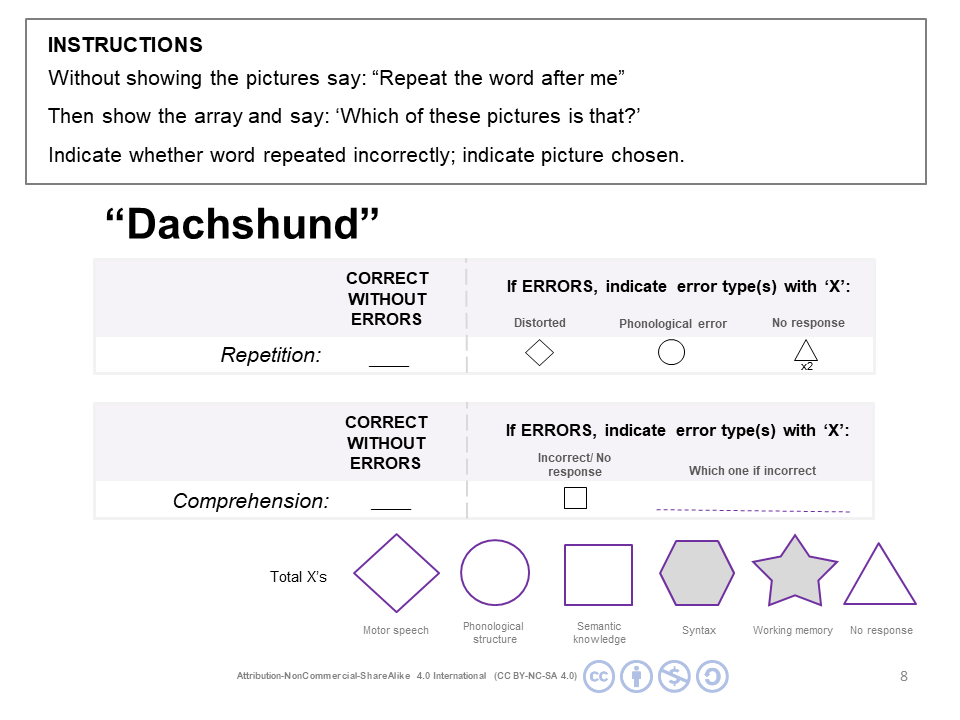

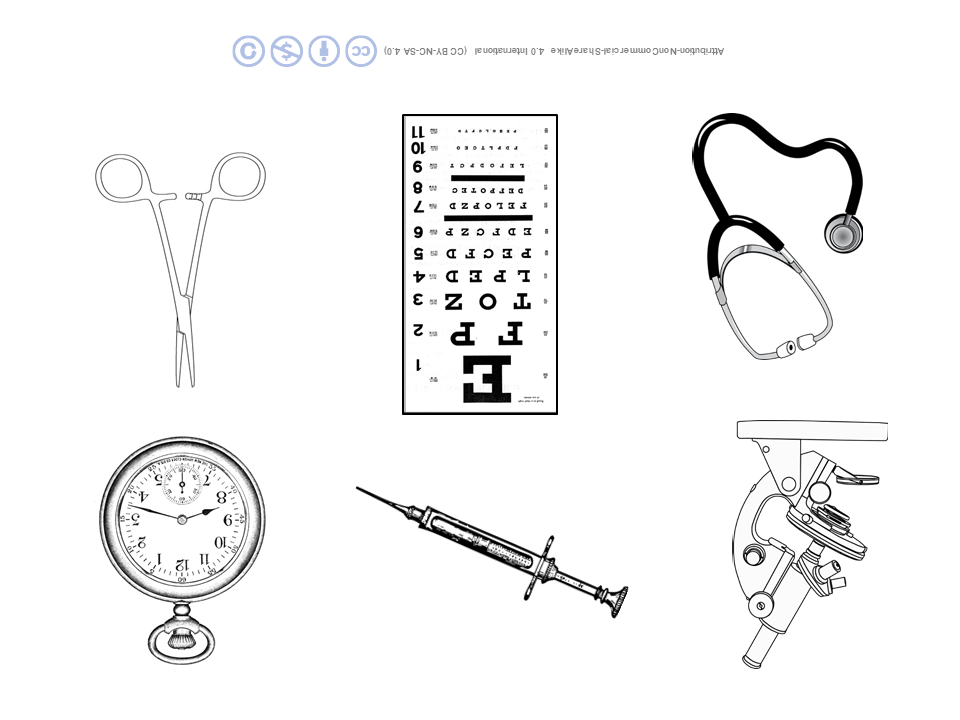

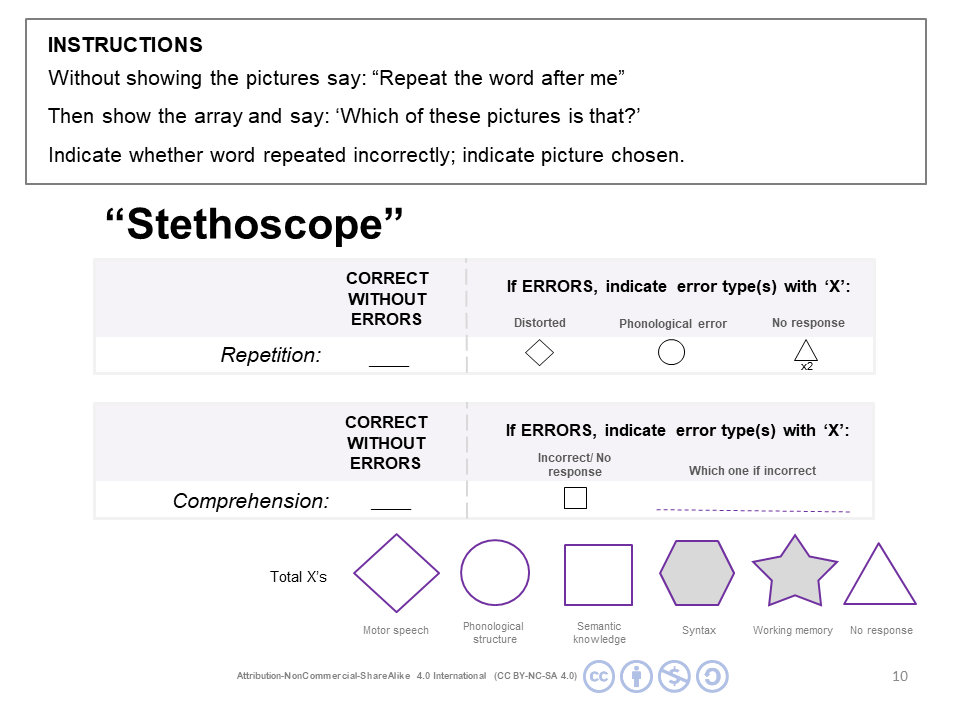

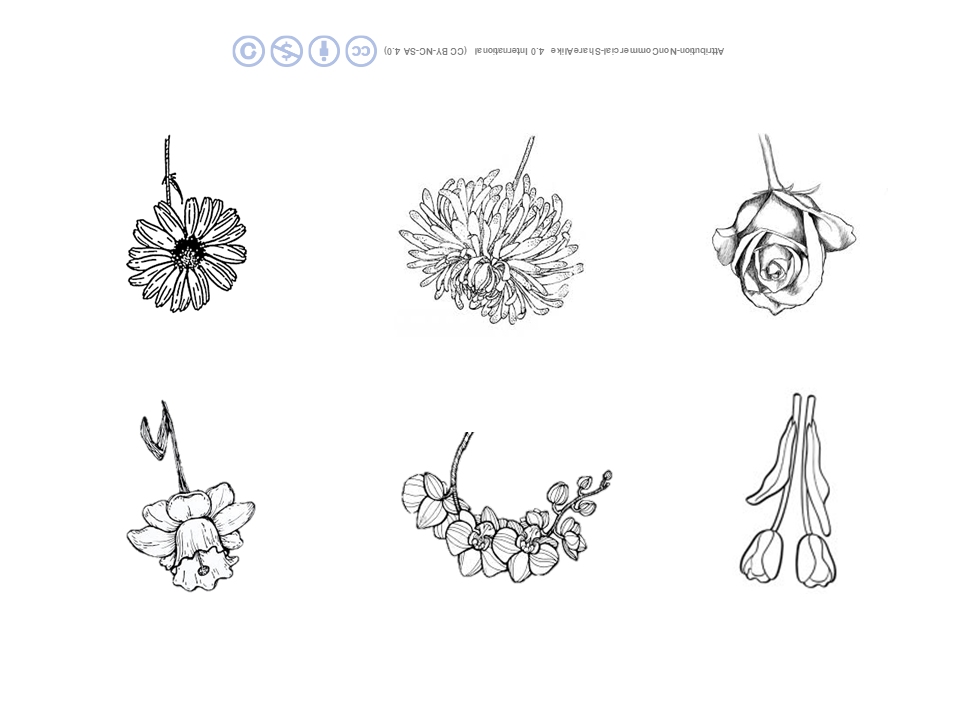

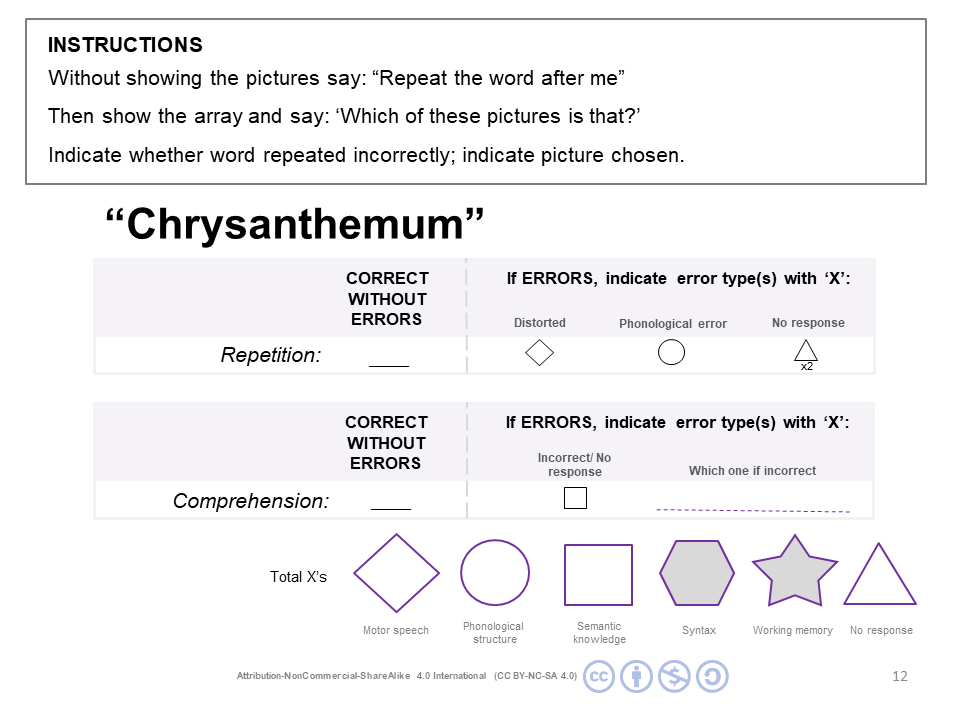

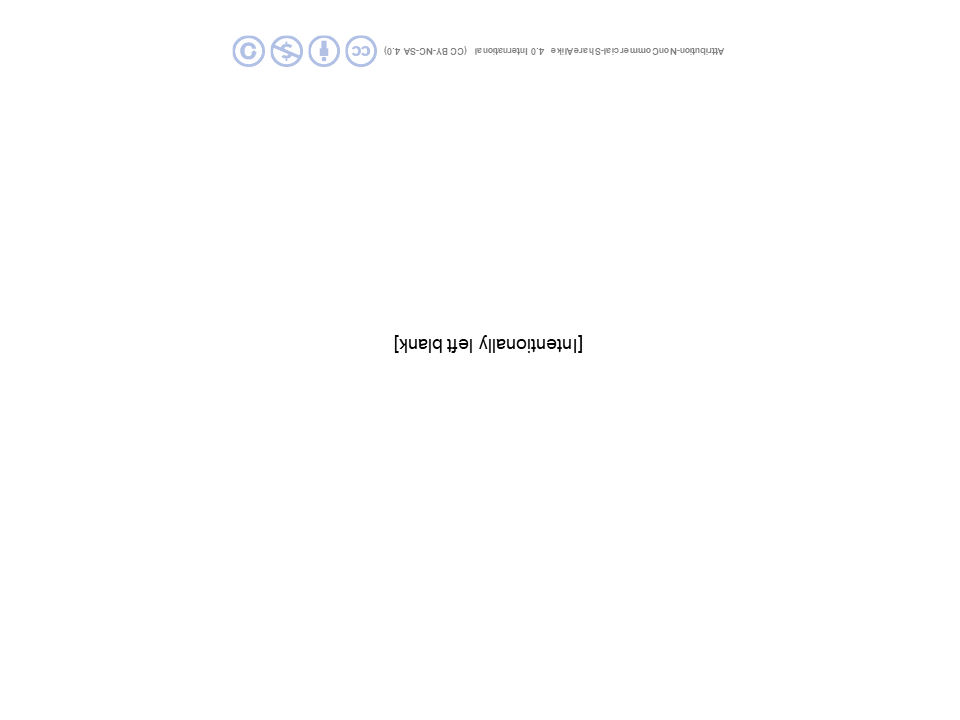

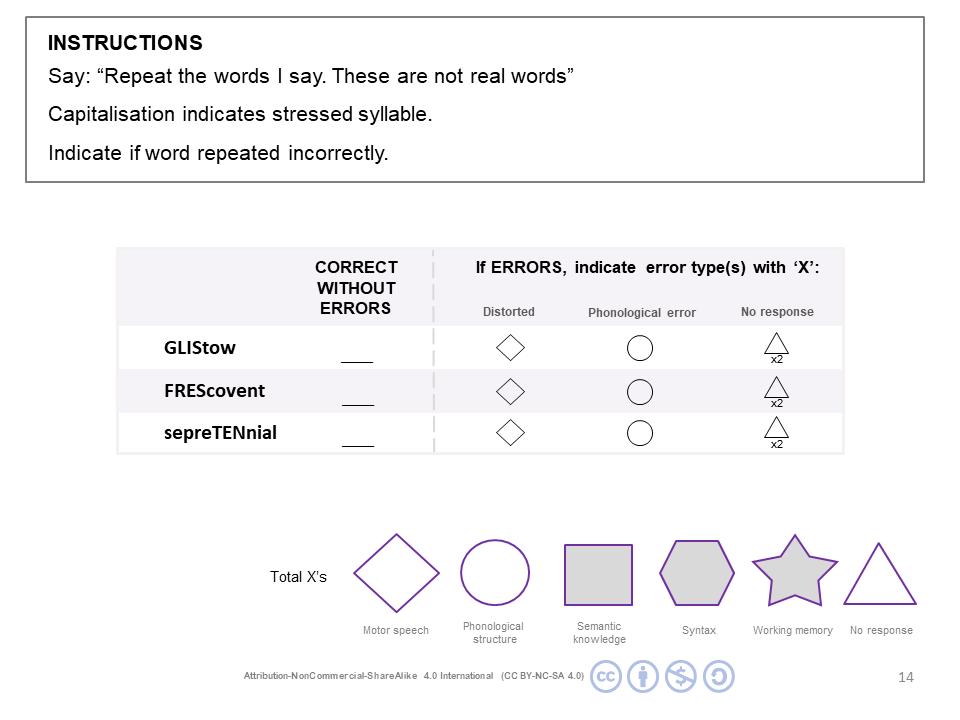

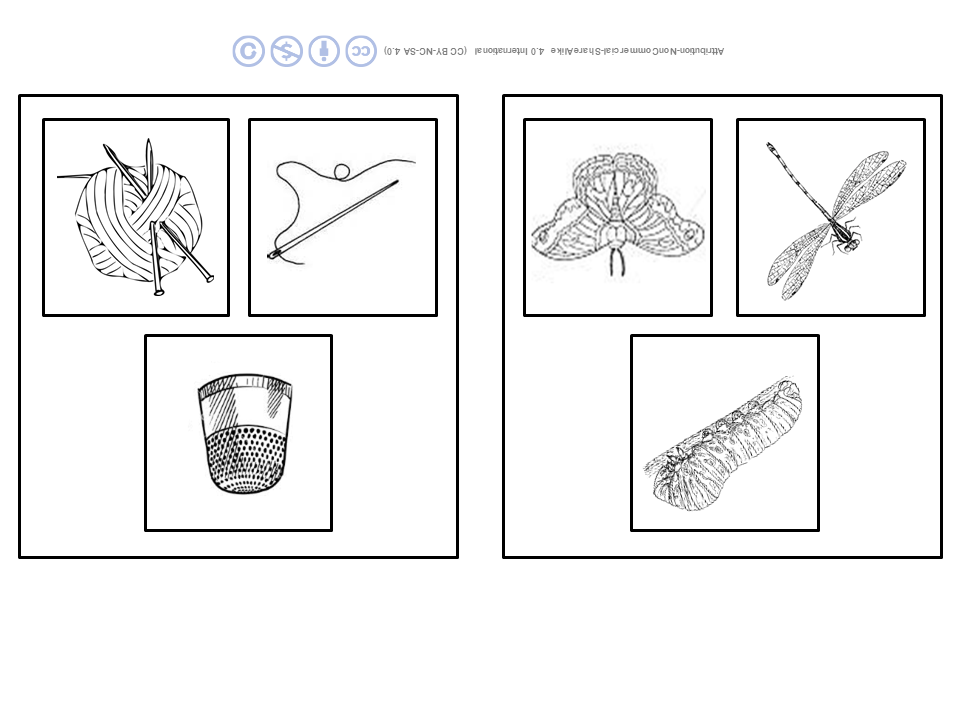

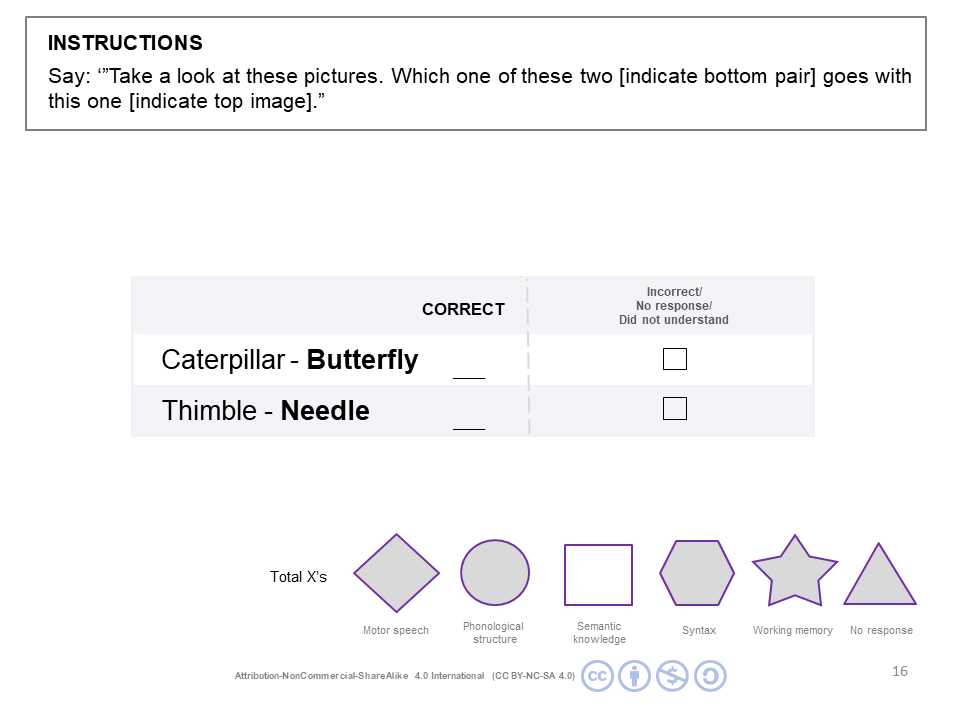

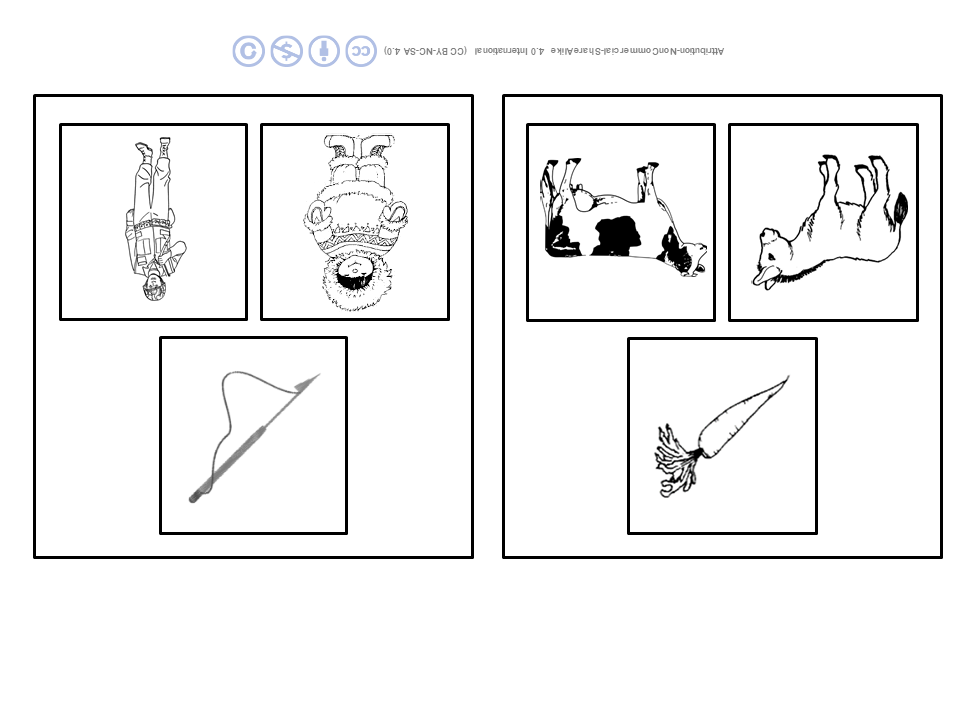

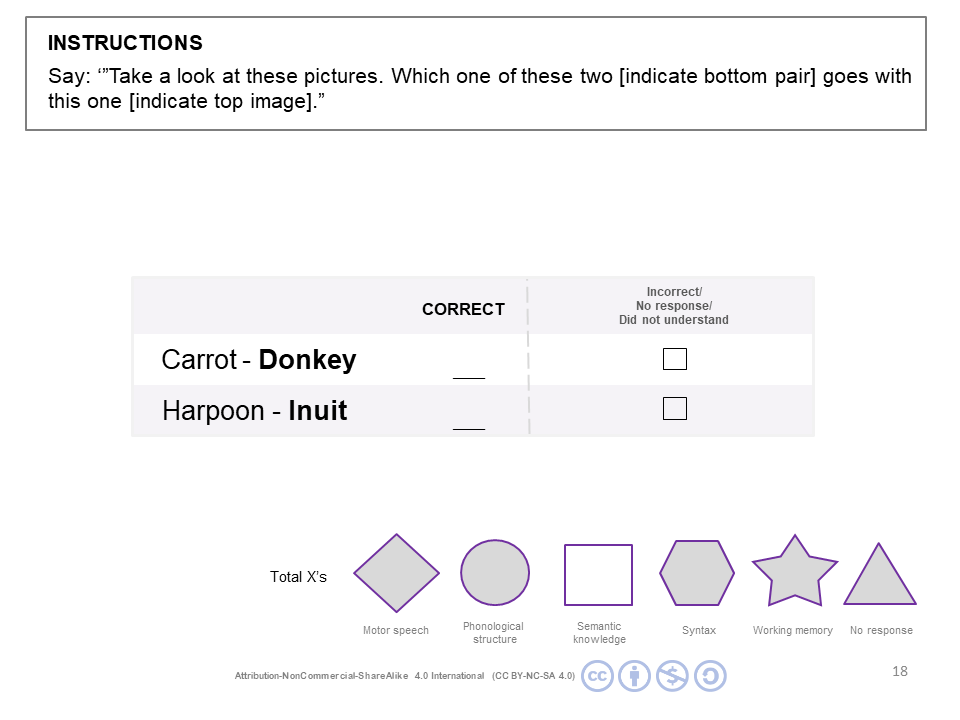

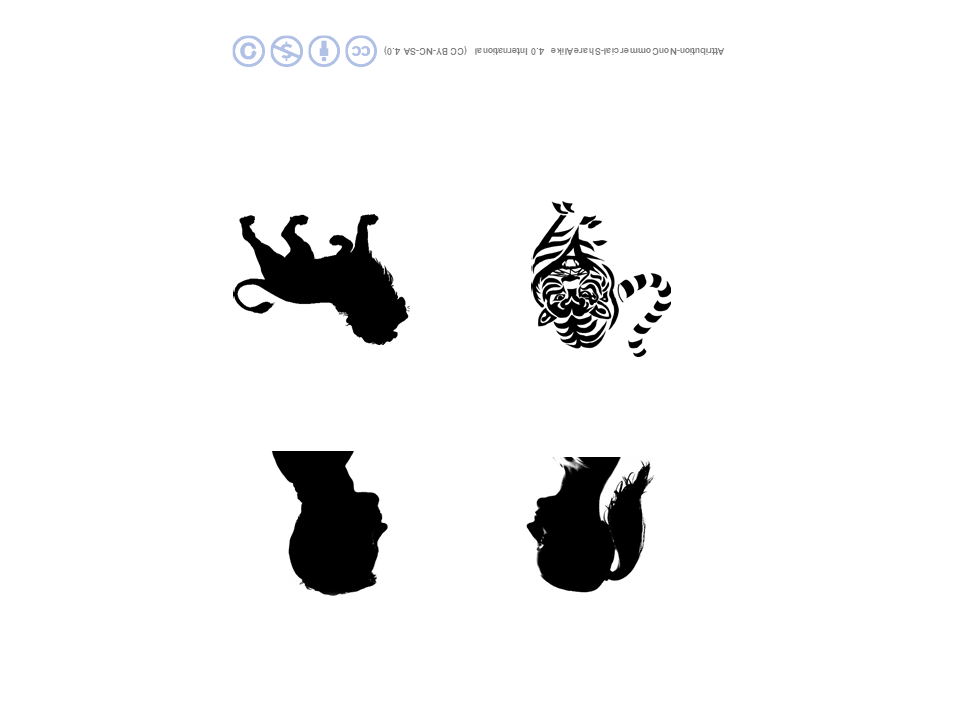

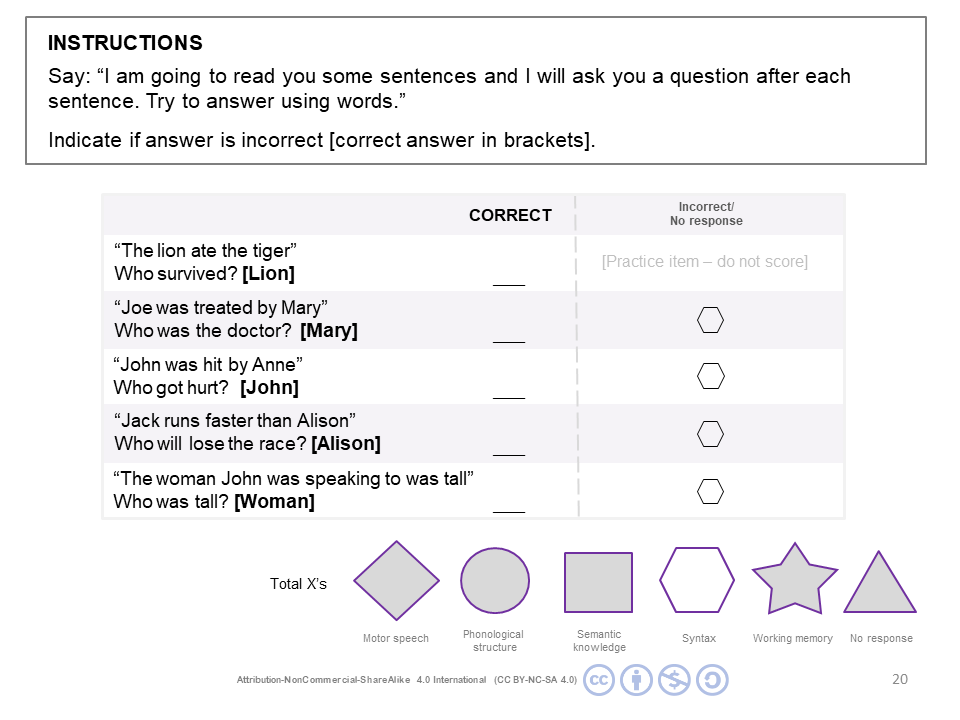

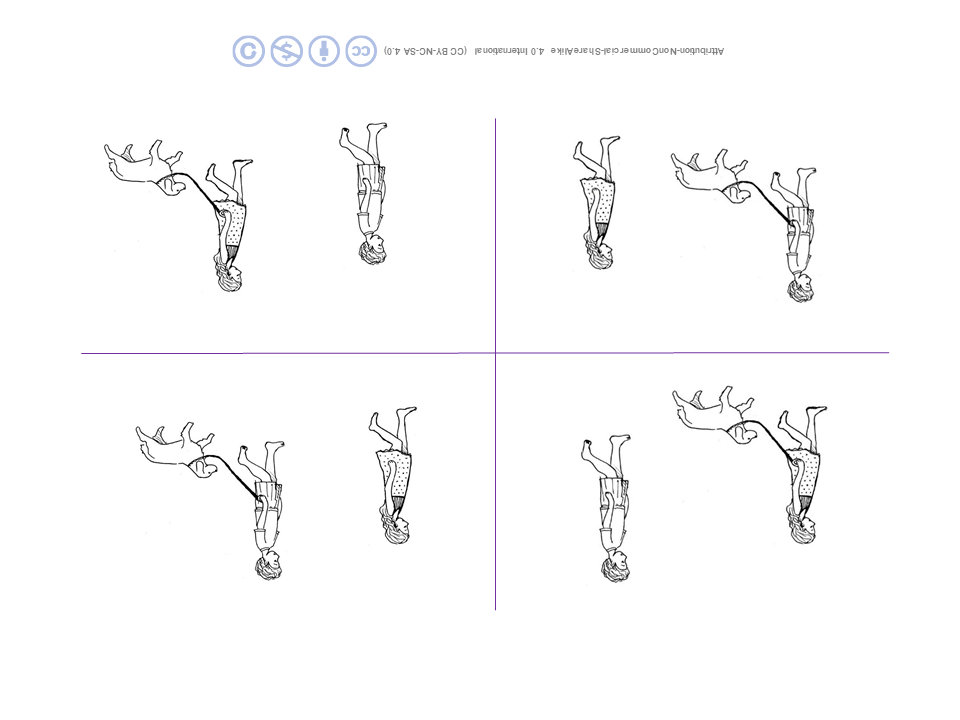

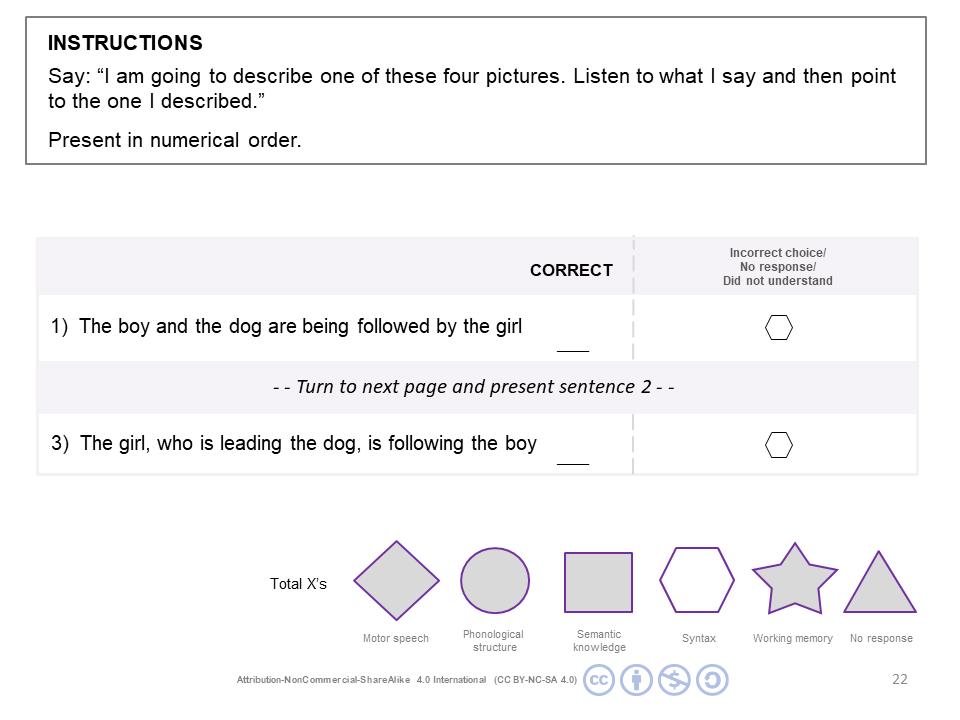

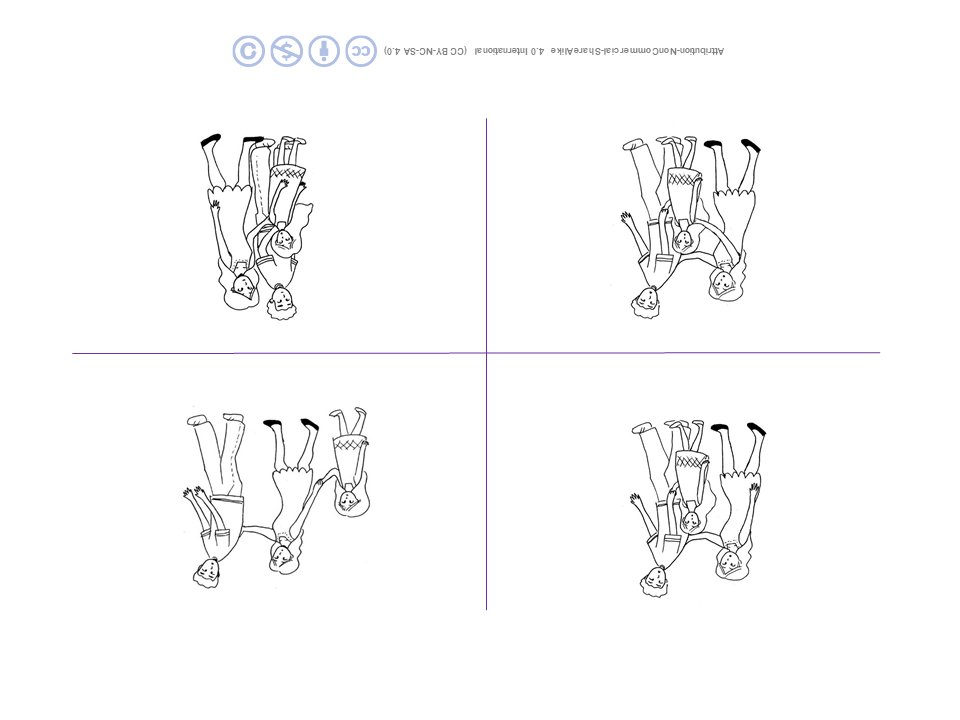

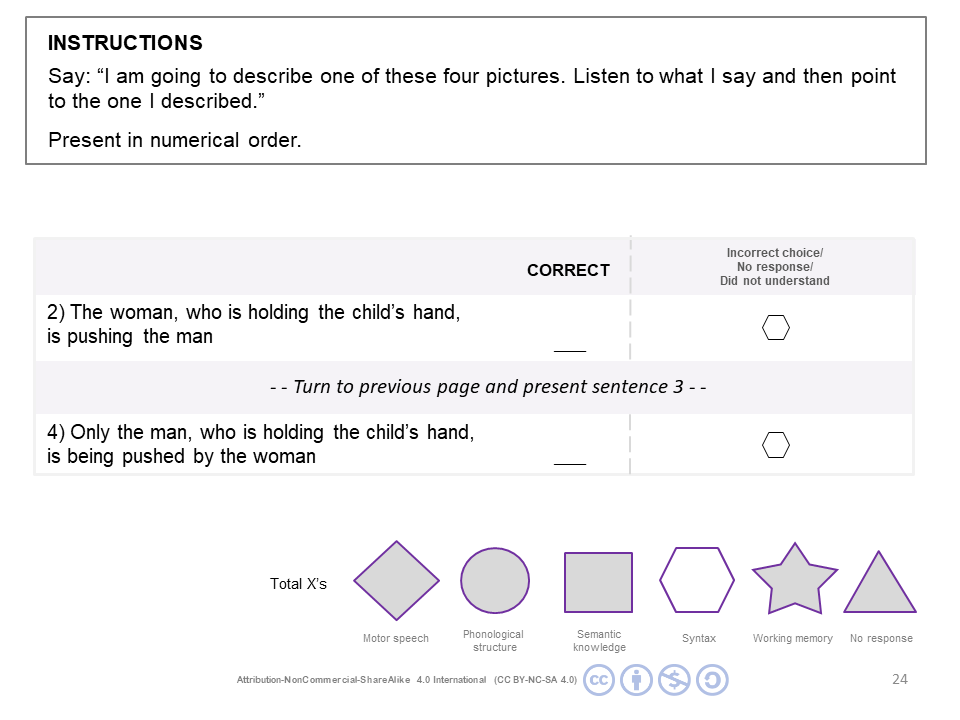

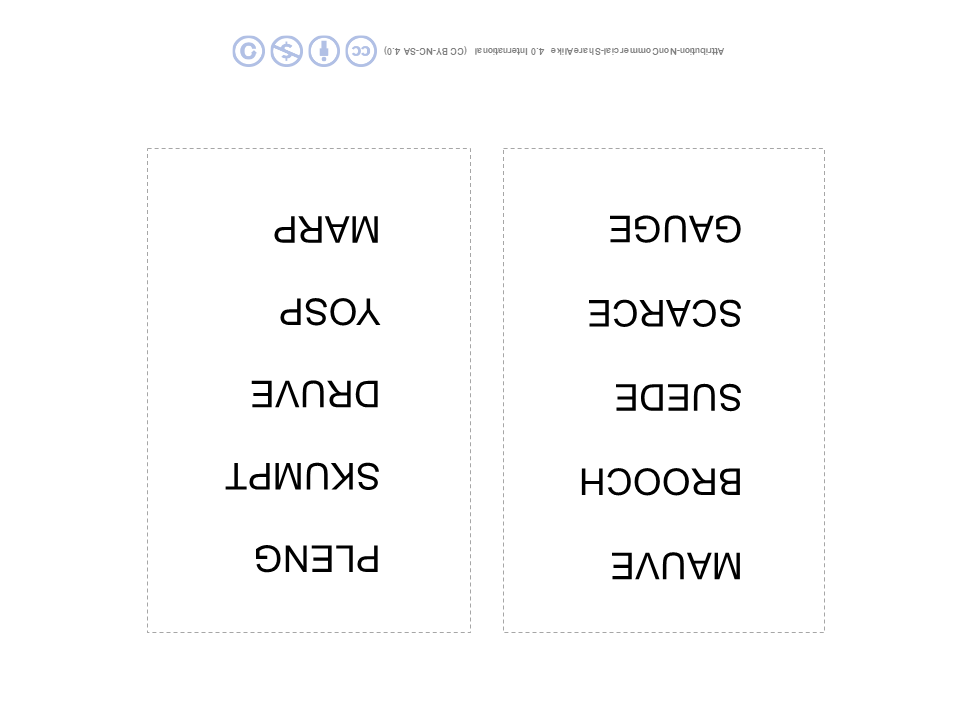

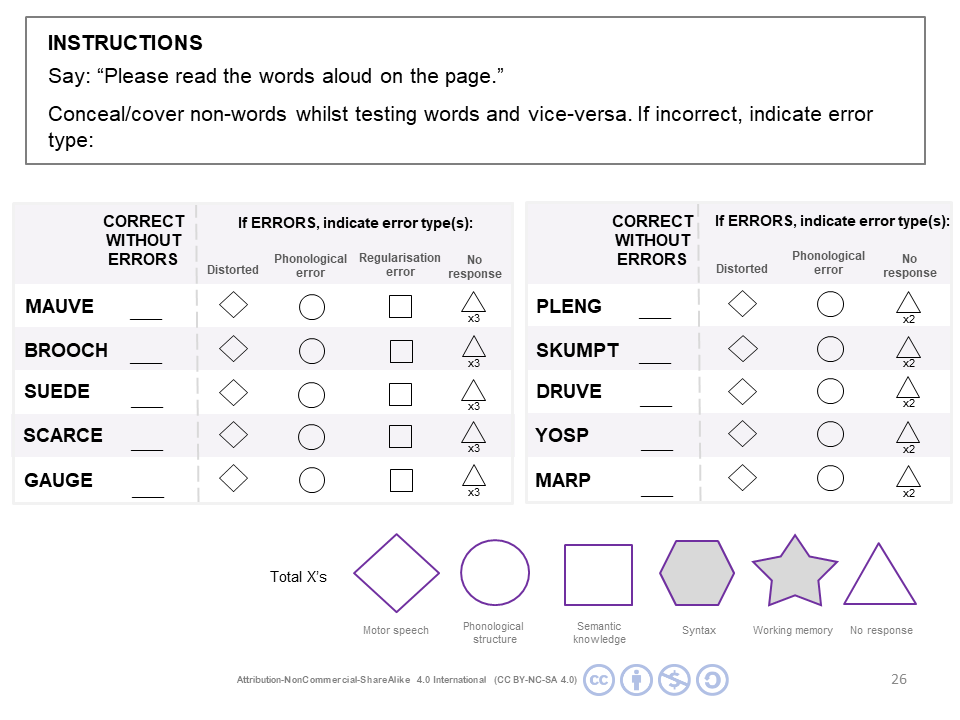

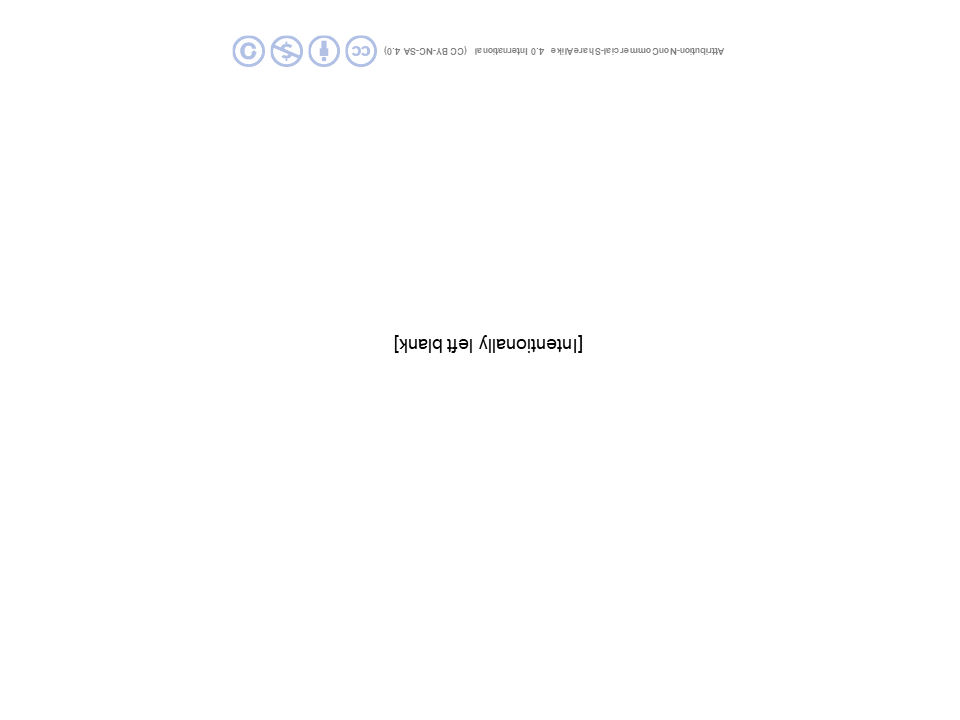

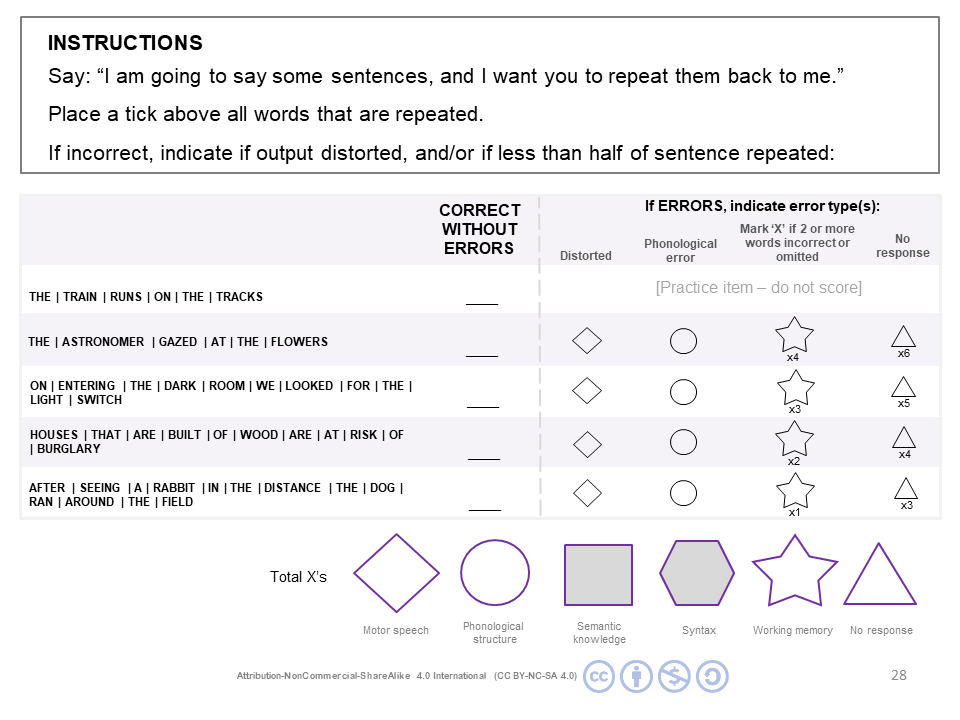

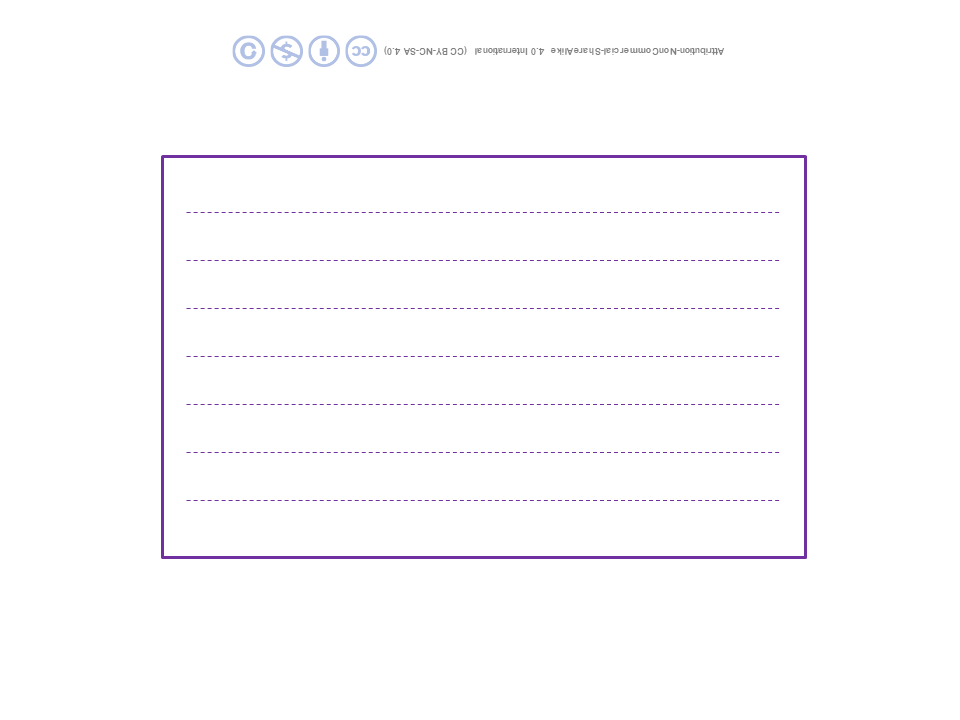

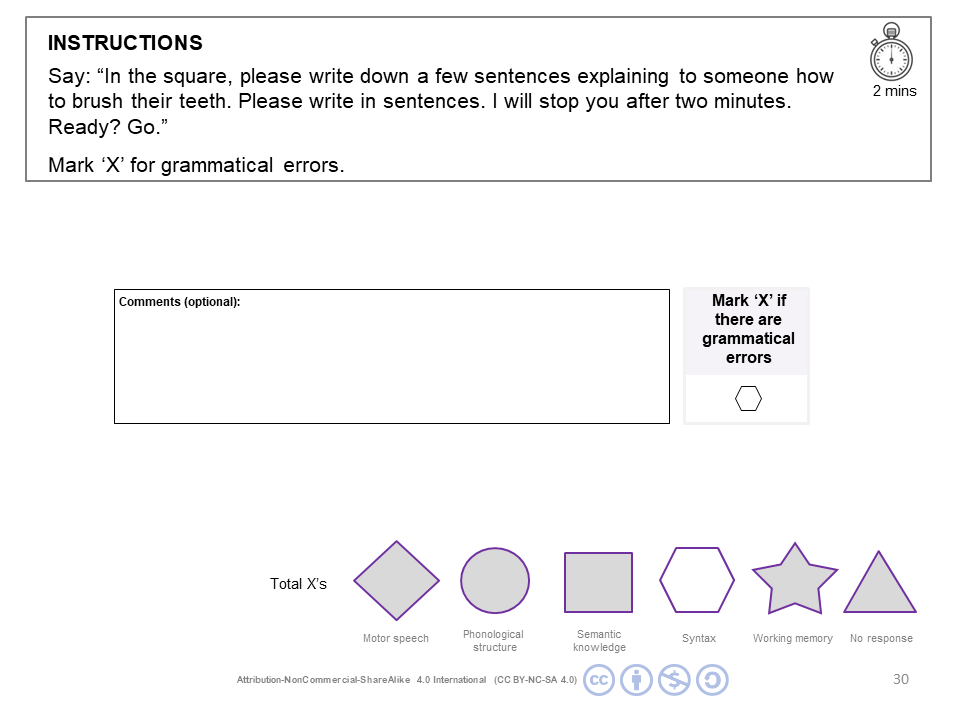

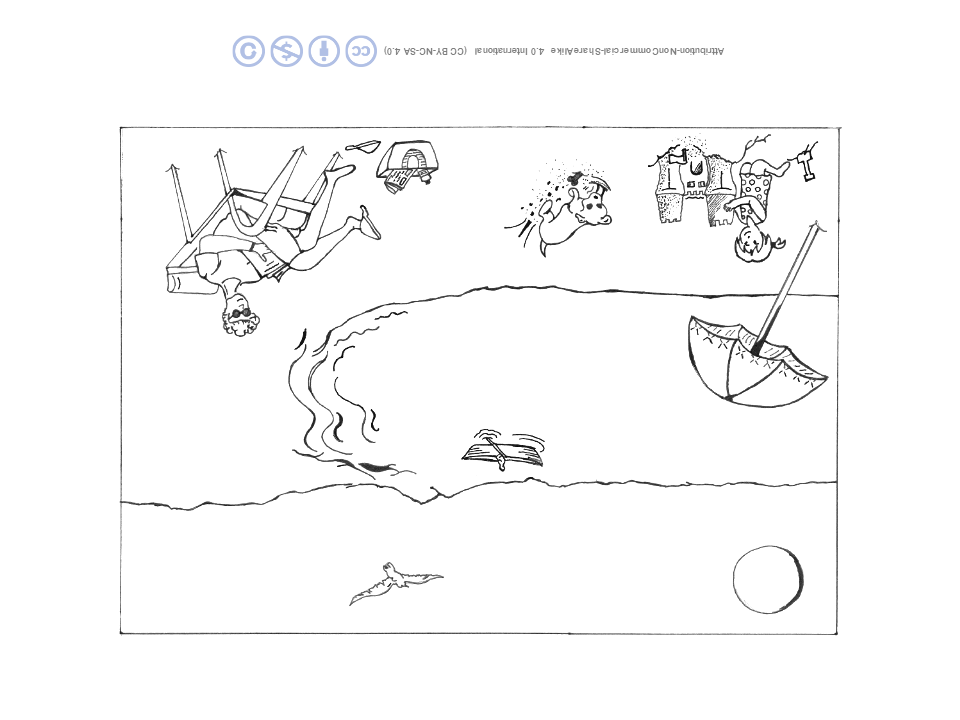

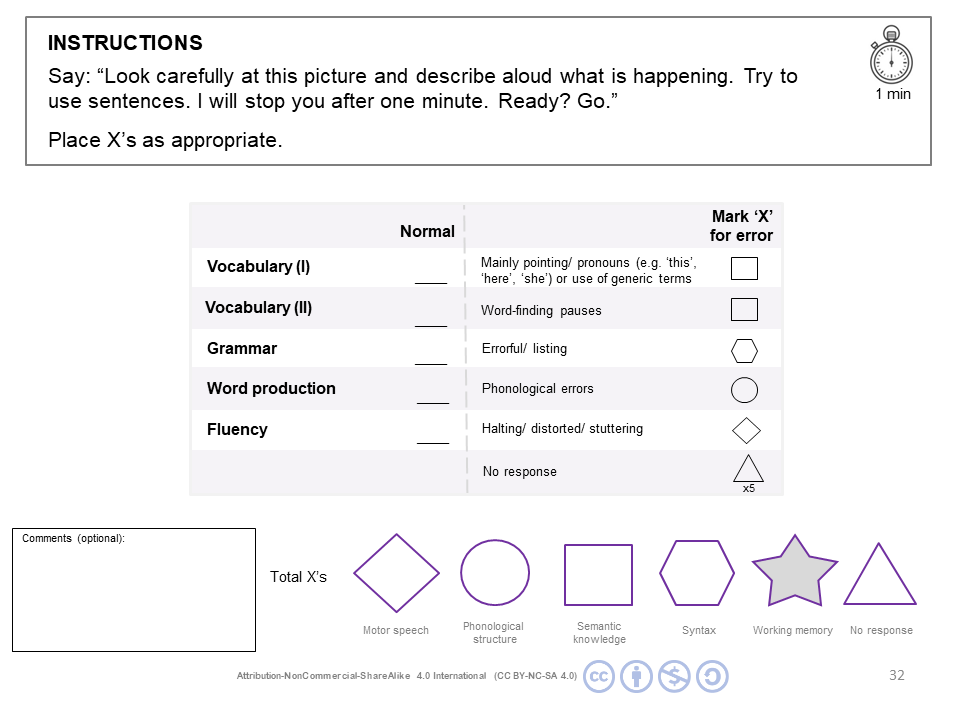

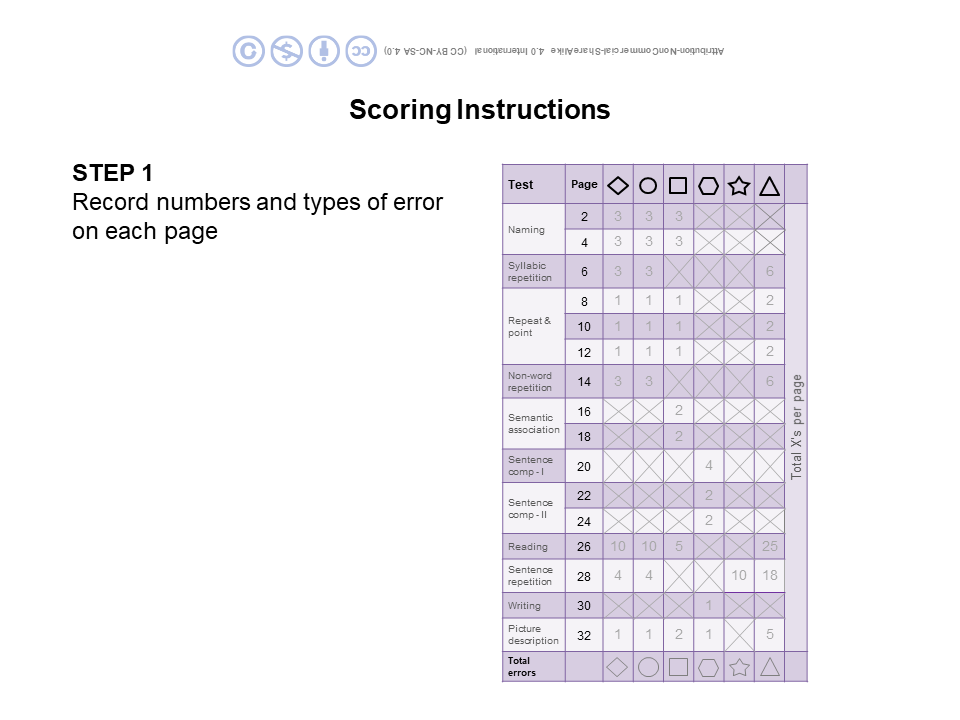

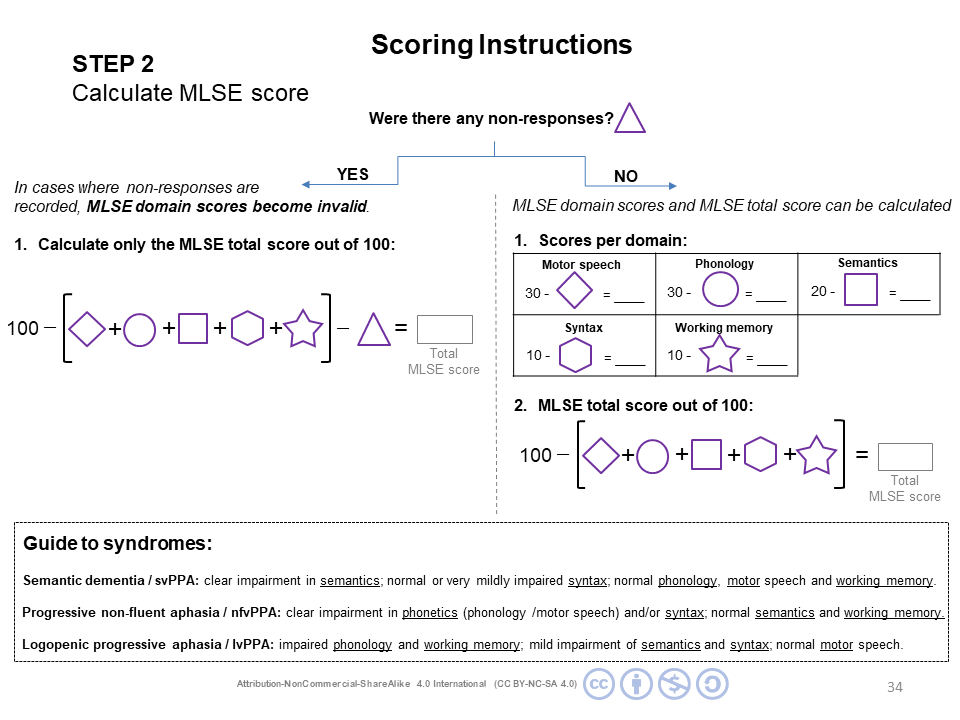

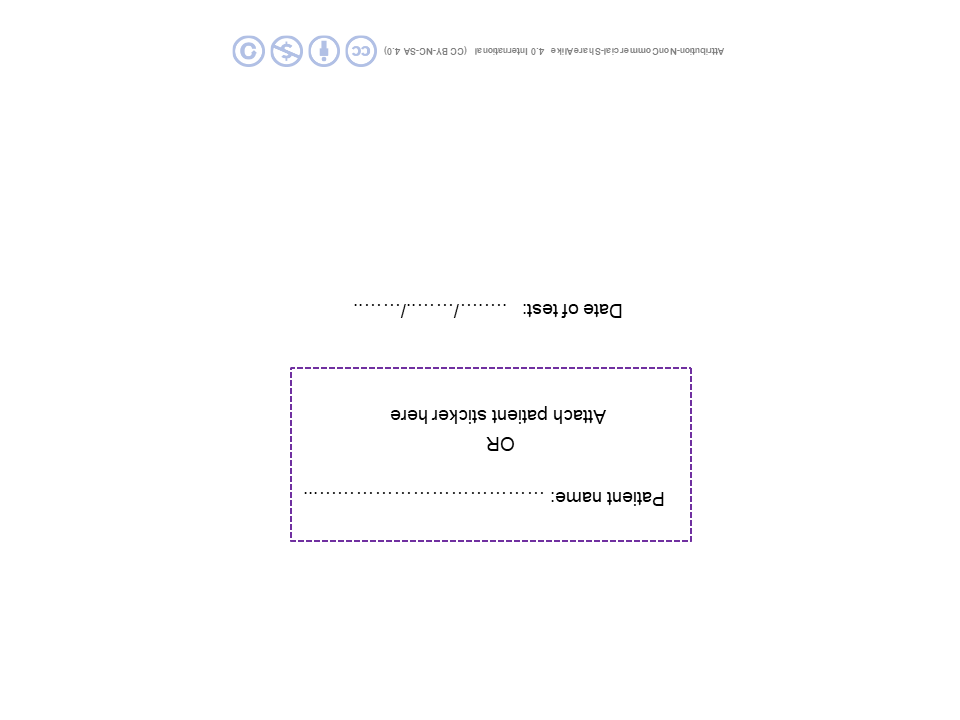


**
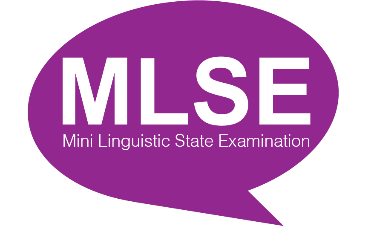
**

**The Mini Linguistic State Examination (MLSE)**

Administration and scoring guide for the English language version

# Introduction

The Mini Linguistic State Examination (MLSE) is designed to be a brief yet comprehensive test of linguistic ability for use in the assessment, clinical classification, and monitoring of progressive aphasic syndromes. The MLSE is a multi-lingual test that was initially developed and validated for use in English- and Italian-speaking populations, but is capable of being adapted for other languages and cultures. This document provides a guide for the administration and scoring of the English-language version of the MLSE. Please familiarise yourself with the test instructions before administering the MLSE. For other language-specific administration and scoring guides, please see our website (<http://www.mlsexam.com>).

# The MLSE booklet

The MLSE takes the form of a test booklet which is accessible via the MLSE website (<http://www.mlsexam.com>), along with updates on publications and additional language-specific versions of the test. The booklet contains stimuli and score sheets on facing pages, designed so that the stimulus sheets face the participant, and the score sheets face the tester. The booklet should therefore be positioned on a surface separating tester and participant.

# Administration and scoring

Administration of the MLSE test takes on average 20 minutes, with a further 5 minutes required to calculate the score. The MLSE features 11 subtasks, which correspond to the major linguistic domains commonly affected in primary progressive aphasia (Gorno-Tempini *et al.*, 2011). These 11 subtasks are considered as encompassing more fundamental functional domains of language production and comprehension, namely: motor speech; semantic knowledge; phonology; syntax and working memory. The scoring of the test aims to capture the nature of a participant’s language impairment by counting the number of errors that correspond to each of these domains, providing a five-dimensional profile score. Brief definitions of the five possible error types are provided below in Supplementary Table 1, and more detailed explanations, with examples, are given for each subtask. A separate overall score out of 100 can also be calculated. Multiple error types can be made on a single item: a semantic error on naming that was phonologically incorrect would indicate that both a semantic and a phonological error had been made.

**Supplementary Table 1.** General definitions of the five types of errors that can be counted during administration of the MLSE.

| **Error type** | **Definition** | **Notes** |
| --- | --- | --- |
| **Motor speech** | A response that is rather slurred, stuttered or contorted, and which the examiner would find somewhat difficult to repeat or transcribe. | Motor speech errors arise only during tasks requiring speech production.  **Any** motor speech error should be noted and scored, even when self-corrected. |
| **Phonological** | A response that contains incorrect but word-like components, and which could easily be repeated or written down. | Phonological errors arise only during tasks requiring speech production.  **Any** phonological error should be noted and scored, even when self-corrected. |
| **Semantic** | A semantic error is noted when a participant’s response suggests a deficit at the level of conceptual knowledge and/or word meaning. | Semantic errors can arise during both production (e.g. naming) and comprehension (e.g. picture association) tasks. Context-specific guidance is provided for each subtask. |
| **Syntactic** | A syntactic error occurs when a participant demonstrates difficulty understanding or producing grammatically correct sentences. | Context-specific guidance is provided for each subtask. |
| **Working memory** | Working memory errors are recorded when a participant is unable to repeat sentences correctly. The shorter the incorrectly repeated sentence, the higher the error score. | Working memory errors are scored only during the sentence repetition task. |

To maximise the potential for profiling the participant according to his/her linguistic domain scores, it is important to try, throughout the test, to avoid using the ‘no response’ error category (marked in the score sheet as triangles). **Therefore, participants should be encouraged to guess throughout the test if they are unsure**. If a *no response* error does occur (e.g. if any of the triangles is ticked), its value is the sum of all possible domain error scores for that item (see Supplementary Figure 1 below).


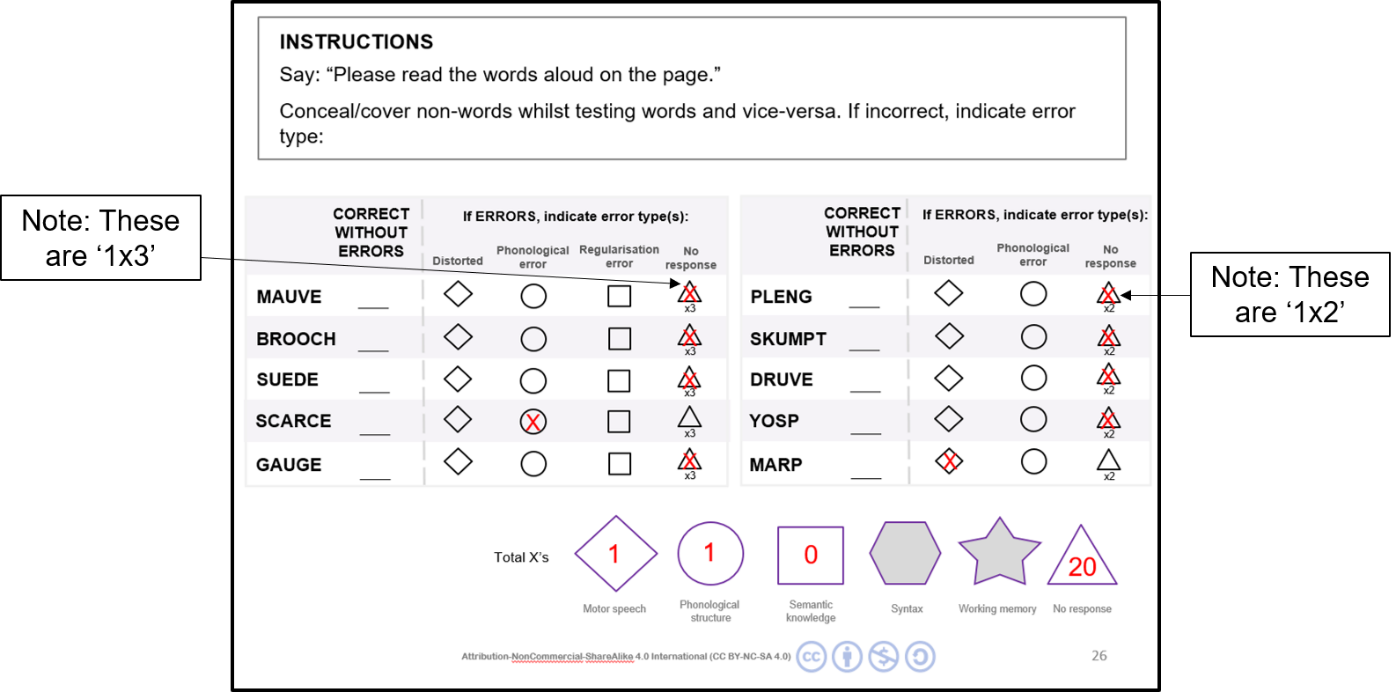


**Supplementary Figure 1.** Example calculation of ‘no response’ errors for the word reading exercise. NB: an ‘X’ in any triangle is equal to the number of possible domain errors in the same row. In this example, for the irregular words on the left, there are four non-responses each of which is multiplied by 3 (4x3 = 12). For the non-words on the right, there are also four non-responses each of which is multiplied by 2 (4x2 = 8). Therefore, the total ‘no response’ errors on this page is 20 (12+8).

Detailed administration and scoring instructions are provided below for each subtask in the order in which they appear in the test. See section 5 for instructions on how to administer and score if a participant has severe speech production impairment. If possible, leave the scoring of total errors per page (marked in the shapes at the bottom of each page) until the end of the session to avoid participant anxiety, which may negatively influence performance on the test.

## Picture naming

**Administration:** There are three picture stimuli per page. Present each picture individually (use pieces of card or your hands to cover the other pictures). Ask the participant to name the object. If the participant: fails to produce the target word after a delay; indicates that they cannot think of the word; or provides an incorrect but semantically related word, then give the phonological cue supplied on the score sheet.

**Scoring:** If the response matches the target (before or after the phonological cue) and contains no phonological or motor speech errors, tick “Correct without errors”. If a motor speech, phonological or semantic error occurs, then place a cross in all the corresponding boxes.

**Error types**: Errors are identified in accordance with the guidelines in Supplementary Table 2.

**Supplementary Table 2.** Guide to different types of picture naming errors

| **Motor Speech** | The following examples should be recorded as motor speech errors:  (i) A spoken response which is rather slurred, stuttered or contorted and which the examiner would find somewhat difficult to repeat or transcribe.  (ii) A written response (in the case of severe speech production impairment)  **Note: any motor speech error should be noted, whether it occurs before or after the phonological cue is provided, and if it is self-corrected.** |
| --- | --- |
| **Phonological Structure** | A response which contains incorrect but word-like components, and which could easily be repeated or written down. Phonological errors include substitutions (e.g. “corksquig” for Corkscrew), repetitions (e.g. “co-corkscrew” for Corkscrew), and omissions (e.g. “corkcrew” for Corkscrew) of speech sounds.  Distinct from post-cue neologisms (see definition under semantic knowledge errors).  **Note: any phonological error should be noted, whether it occurs before or after the phonological cue is provided, and if it is self-corrected.** |
| **Semantic Knowledge** | A point is deducted from the Semantic Knowledge domain if a participant fails to provide the target word following the phonological cue.  The following post-cue responses count as ‘Failure to provide the target word following the cue’:  1. Omissions – *“don’t know”, “can’t remember”, or failure to generate a response.*  2. Semantic errors - *replacing the target word with another word which has a different but similar meaning (e.g. “cork” for Corkscrew).*  3. Category only responses - *giving the category superordinate instead of the target word (e.g. “fruit” for Pineapple/ “animal” for Giraffe or Squirrel)*  4. Circumlocutions - *a description of the target, which can be either informative or uninformative, but which does not include the target word. For example, “it’s cold”/ “it’s made of ice” for Igloo, or “I know what it is. I’ve seen one before on holiday” for Giraffe.*  5. Neologisms – *rarely, patients with a semantic impairment may produce a neologism in response to the phonological cue. A neologism is typically defined as a non-word which shares less than 50% of the phonology of the target word (e.g.”eggnut” for Igloo), however, in this case 50% of the phonology of the target word might be preserved when the neologism follows (and incorporates) the phonological cue (e.g. “squiff” produced after the phonological cue for Squirrel), additionally the response may be a real word but semantically unrelated to the target (e.g. “squish” produced after the phonological cue for Squirrel). These responses differ from phonological errors in that they are intentionally produced and not due to production impairment, whereas phonological errors are unintentional errors characterised by repetition, omission or substitution of speech sounds.* |

## Syllable and polysyllabic word repetition (‘pa-ta-ka’)

**Administration:** Instruct the participant that they will be repeating what you say, then read aloud each item (single syllables, polysyllabic word and repeated polysyllabic word). If the participant explicitly requests that a to-be-repeated item is provided again (e.g. because they did not hear), then do so, but no more than once for each test item.

**Scoring:** If the response contains no errors, tick “Correct without errors”. If a motor speech or phonological error occurs, then place a cross in all the corresponding boxes.

**Error types**: Errors are identified in accordance with the guidelines in Supplementary Table 3.

**Supplementary Table 3.** Scoring guide for syllabic and polysyllabic word repetition errors

| **Motor Speech** | A response which is rather slurred, stuttered or contorted and which the examiner would find somewhat difficult to repeat or transcribe.  **Note: a motor speech error should be noted even if it is self-corrected.** |
| --- | --- |
| **Phonological Structure** | A response which contains incorrect but word-like components, and which could easily be repeated or written down. Phonological errors include substitutions (e.g. “catalippa” for Caterpillar), repetitions (e.g. “ca-caterpillar” for Caterpillar), and omissions (e.g. “pillar” for Caterpillar) of speech sounds.  **Note: a phonological error should be noted even if it is self-corrected.** |
| **No Response** | (i) No response after encouragement to guess.  (ii) Subtest/item not administered or attempted. |

## Repeat and point

**Administration:** Cover, or otherwise conceal, the array of pictures facing the participant, and instruct the participant to “repeat this word after me”. Read the stimulus word aloud. The tester may present the stimulus word again if explicitly requested by the subject, but not more than once for each test item. Following a response from the participant, uncover the array of pictures and instruct the participant to point to the picture that matches the word. If the participant has not responded after 5 seconds, repeat the instruction. Encourage participant to guess if they indicate that they are unsure.

**Scoring:** For repetitions with no errors and correct picture-matching responses tick “Correct without errors”. If a motor speech, phonological or semantic error occurs, then place a cross in all the corresponding boxes.

**Error types**: Errors are identified in accordance with the guidelines in Supplementary Table 4.

**Supplementary Table 4.** Scoring guide for repeat and point errors

| **Motor Speech** | A response which is rather slurred, stuttered or contorted and which the examiner would find somewhat difficult to repeat or transcribe.  **Note: a motor speech error should be noted even if it is self-corrected.** |
| --- | --- |
| **Phonological Structure** | A response which contains incorrect but word-like components, and which could easily be repeated or written down. Phonological errors include substitutions (e.g. “tessascope” for Stethoscope), repetitions (e.g. “ste-stethoscope” for Stethoscope), and omissions (e.g. “stethscope” for Stethoscope) of speech sounds.  **Note: a phonological error should be noted even if it is self-corrected.** |
| **No Response (Repetition)** | (i) No response after encouragement to guess.  (ii) Subtest/item not administered or attempted. |
| **Semantic Knowledge** | For the ‘point’ component of this task, an error is noted if participant selects an incorrect item or provides no response. |

## Non-word repetition

**Administration:** Instruct the participant that they will be repeating what you say, then read aloud each non-word. Pronunciation is indicated by capitalisation of the stressed syllable. If necessary, remind the participant to repeat what was just said. Re-presentation of an item by the tester is allowed only once, if explicitly requested by the subject.

**Scoring:** If the response contains no errors, tick “Correct without errors”. If a motor speech or phonological error occurs, then place a cross in all the corresponding boxes.

**Error types**: Errors are identified in accordance with the guidelines in Supplementary Table 5.

**Supplementary Table 5.** Scoring guide for non-word repetition errors

| **Motor Speech** | A response which is rather slurred, stuttered or contorted and which the examiner would find somewhat difficult to repeat or transcribe.  **Note: a motor speech error should be noted even if it is self-corrected.** |
| --- | --- |
| **Phonological Structure** | A response which contains incorrect but word-like components, and which could easily be repeated or written down. Phonological errors include substitutions (e.g. “bescavent” for Frescovent), repetitions (e.g. “fres-frescovent” for Frescovent), and omissions (e.g. “fresowent” for Frescovent) of speech sounds.  **Note: a phonological error should be noted even if it is self-corrected.** |
| **No Response** | (i) No response after encouragement to guess.  (ii) Subtest/item not administered or attempted. |

## Semantic association

**Administration:** There are four test items, which are groups of three pictures (indicated by a box surrounding each group). Present each group of images individually (covering the other one on the page). Instruct the participant to indicate ‘which of the two pictures on the bottom row [point to the bottom row] ‘goes with’ the one above [point to the top row]’. Encourage participant to guess if they indicate that they are unsure.

**Scoring:** If the response matches the target tick “Correct”.

**Error types**: If the wrong image is chosen, no response given, or the participant did not understand the task, place an ‘X’ in the box for semantic knowledge error.

## Sentence comprehension (i)

**Administration:** Instruct the participant that they are to listen to a statement and then answer a question. The participant can answer the question by using a word or by pointing to one of the four pictures on the stimulus side of the booklet. The pictures represent a lion and a tiger (to represent the nouns in the first sentence, which is a practice item and not scored) and a male and female head (to stand for the masculine and feminine names in the remaining sentences). The male/female heads should be covered during presentation of the practice item, and the lion/tiger during presentation of the test items. The correct answer is indicated in brackets on the score sheet. Repetition of a sentence by the tester is allowed only once, and only if explicitly requested by the subject.

**Scoring:** If the response matches the target tick “Correct”.

**Error types**: If the participant gives the wrong answer or no response, place an ‘X’ in the box for syntax error.

## Sentence comprehension (ii) with picture stimuli

**Administration:** Instruct the participant that they are to listen to a statement and indicate which of four pictures the statement describes. Re-presentation of a sentence by the tester is allowed only once and only if explicitly requested by the subject. There are four sentences to be matched, and two sets of four pictures, which appear on successive pages. Sentences are numbered: 1 and 3 are on the first page, 2 and 4 on the second. **Ensure that sentences are presented to the subject in numbered order.**

**Scoring:** If the correct image is chosen tick “Correct”.

**Error types**: If the participant makes an incorrect choice, no response, or could not understand the task instruction, place an ‘X’ in the box for syntax error.

## Word and non-word reading

**Administration:** Show the participant the column of irregularly spelled words, while covering the column of non-words, and instruct the participant to read each word aloud. Encourage participant to guess if they indicate that they are unsure. Then cover the column of irregularly spelled words and ask the participant to read aloud the non-words.

**Scoring:** If the response matches the target and contains no errors tick “Correct without errors”. If a motor speech, phonological or regularisation error occurs, then place a cross in all the corresponding boxes.

**Error types**: Errors are identified in accordance with the guidelines inSupplementary Table 6.

**Supplementary Table 6.** Scoring guide for reading errors

| **Motor Speech** | A response which is rather slurred, stuttered or contorted and which the examiner would find somewhat difficult to repeat or transcribe.  **Note: a motor speech error should be noted even if it is self-corrected.** |
| --- | --- |
| **Phonological Structure** | A response which contains incorrect but word-like components, and which could easily be repeated or written down. Phonological errors include substitutions (e.g. “scarf” for Scarce), repetitions (e.g. “sca-scarce” for Scarce), and omissions (e.g. “scare” for Scarce) of speech sounds.  **Note: a phonological error should be noted even if it is self-corrected.** |
| **Semantic Knowledge** | A *regularisation error* when reading the irregular words: a response in which the participant applies typical pronunciation rules. The regularised pronunciations of the five irregular words are shown in Supplementary Table 7 (with International Phonetic Alphabet [IPA] transcription).  **Note: a regularisation error should be noted even if it is self-corrected.** |
| **No Response** | (i) No response after encouragement to guess.  (ii) Subtest/item not administered or attempted. |

**Supplementary Table 7.** Regularised pronunciations of the irregular words

| **Word** | **Description of the regularised pronunciation (regularisation error)** | **IPA transcription of regularised pronunciation*** |
| --- | --- | --- |
| **Mauve** | Pronounced with an “aw” sound as in ‘saw’ | /mɔv/ |
| **Brooch** | Pronounced with an “oo” sound as in ‘brood’ | /brutʃ/ |
| **Suede** | Pronounced with:  (i) an “ee” sound: ‘swede’  (ii) an “oo” sound as in ‘brood’  (iii) a “yoo” sound as in ‘feud’ | (i) /swid/  (ii) /sud/  (iii) /sjud/ |
| **Scarce** | Pronounced with an “ah” sound as in ‘hard’ | /skɑs/ |
| **Gauge** | Pronounced with:  (i) an “ow” sound: ‘gouge’  (ii) an “aw” sound as in ‘saw’ | (i) /gaɷdʒ/  (ii) /gɔdʒ/ |

*NB: regularisation errors should be noted for responses which contain both a regularisation of the vowel sound and a phonological error (e.g “scarf” for Scarce), and which, therefore, do not exactly match the IPA transcriptions.

## Sentence repetition

**Administration:** Instruct the participant to repeat the sentences that will be read aloud to them. Repetition of a sentence by the tester is allowed only once, and only if explicitly requested by the participant.

**Scoring:** Mark a small tick on the score sheet above each individual word that is repeated correctly in the sentence. If fewer than two words are incorrect (e.g. substituted for a different word or omitted) tick “Correct without errors”. If a motor speech, phonological or working memory error occurs, then place a cross in all the corresponding boxes. NB: If a participant is clearly struggling on this task during the first two sentences, the remaining sentences can be skipped but should be scored in the same way as the first two.

**Error types**: Errors are identified in accordance with the guidelines in Supplementary Table 8.

**Supplementary Table 8.** Scoring guide for sentence repetition errors

| **Motor Speech** | A response which is rather slurred, stuttered or contorted and which the examiner would find somewhat difficult to repeat or transcribe.  **Note: a motor speech error should be noted even if it is self-corrected.** |
| --- | --- |
| **Phonological Structure** | Phonological errors during repetition, e.g. responses which contain incorrect but word-like components, and which could easily be repeated or written down. Phonological errors include substitutions, repetitions, and omissions of speech sounds.  **Note: a phonological error should be noted even if it is self-corrected.** |
| **Working Memory** | If the participant (i) provides a response, but (ii) two or more words are omitted or are substituted for different words, mark ‘X’ in the corresponding box. |
| **No Response** | (i) No response after encouragement to guess.  (ii) Subtest/item not administered or attempted. |

## Written description

**Administration:** Read the test instruction to the participant. The instruction may be repeated once if the participant explicitly asks, *or* if the participant does not write anything within 10 seconds. If the participant writes very little, or asks something like “is that enough?” within a minute of starting the task, encourage them by saying “Can you add anything more?” Allow the participant to write for two minutes (though do not instruct them to stop in the middle of a sentence or a word).

**Scoring:** If writing is illegible, ask the participant to read back what they have written. If it is clear that their writing sample matches what they read back, grammaticality can be scored based on what they read back. Use comments box for any observations relevant to performance (e.g. ‘severe tremor’, ‘dystonic writing hand’, etc.).

**Error types**: If there are grammatical errors in the response, mark ‘X’ in the box labelled ‘grammatical errors’. If the participant is unable to write anything, they would also incur *grammatical errors.*

## Picture description

**Administration:** Read the test instruction to the participant. The instruction may be repeated once if the participant explicitly asks, *or* if the participant does not say anything within 10 seconds. Allow the participant to describe the picture for one minute, remaining silent throughout, not offering encouragement, agreement or back-channelling (such as “uh huh”, “yes”, “OK” etc.) If a participant stops before the minute is up, the tester should prompt them to continue by saying “Anything else?” but no additional prompts should be given. If the participant asks for information or clarification during the test, this should be responded to as briefly as possible. After one minute, the tester may stop the participant.

**Scoring:** Features of speech (vocabulary, grammar, word production, and fluency) are categorised according to whether they are “normal” or contain errors. Mark ‘X’ in the appropriate boxes if errors with vocabulary, grammar, word production and/or fluency are detected.

**Error types**: Errors are identified in accordance with the guidelines in Supplementary Table 9.

**Supplementary Table 9.** Scoring guide for picture description errors

| **Motor Speech** | Motor speech is represented by the “Fluency” category in this task. The following examples should be recorded as motor speech errors:  (i) Impaired fluency as denoted in the test manual (halting, distorted or stuttering speech).  (ii) A written response (in the case of severe speech production impairment). |
| --- | --- |
| **Phonological Structure** | Phonological structure is represented by the “Word Production” category. Mark an ‘X’ for impaired Word Production if the participant makes any phonological errors (including self-corrected phonological errors) when describing the picture, (e.g. responses which contain incorrect but word-like components, and which could easily be repeated or written down, including substitutions, repetitions, and omissions of speech sounds). |
| **Semantic Knowledge** | Semantic knowledge is represented by the “Vocabulary” categories.  Vocabulary (I): Mark an ‘X’ for impaired Vocabulary (I) if the participant mainly points or uses pronouns (e.g. “this”, “here”, “she”) or if the participant uses generic terms (e.g. “the dog is playing” instead of “the dog is digging” or “the boy is building something” instead of “the boy is building a sandcastle”).  Vocabulary (II): Mark an ‘X’ for impaired Vocabulary (II) if the participant displays word-finding pauses (e.g. stops to think of a word which they may or may not remember). |
| **Syntax** | Syntax is represented by the “Grammar” category. Mark an ‘X’ for impaired Grammar if the participant:  (i) Makes grammatical errors (e.g. “Dog is digging. Bird is flying.”, “The castle will probably get kicked down than the water”), or  (ii) Lists elements of the picture instead of speaking in sentences. |
| **No Response** | (i) No response after encouragement.  (ii) Subtest/item not administered or attempted. |

# Calculating the score

Performance on the MLSE can be scored according to five linguistic domains and/ or out of an overall score of 100. Scoring according to the linguistic domains can help to provide an indication of the pattern of language impairment exhibited by a patient, and thus to classify patients with different subtypes of aphasia. Calculation of an overall score out of 100, on the other hand, may be more useful for screening or monitoring purposes.

**The ‘No response’ (∆) category:** Participants should always be encouraged to guess if they are unsure of any of the test items to avoid non responses. If any ‘triangles’ are ticked, domain scores become invalid. In this case, **only the Total Score should be used**. It is therefore encouraged to force a guess to avoid ‘no responses’ so that the domain scores can be used. Note again that the value of any ticked ‘no response’ triangle is equal to the total number of possible domain errors in its row (see Supplementary Figure 1 above).

**Scoring according to the linguistic domains:** Scores for the linguistic domains are calculated by summing the number of errors in each category, and subtracting these from the maximum score for the corresponding linguistic domain. The maximum scores for each domain are as follows: motor speech = 30; phonology = 30; semantic knowledge = 20; syntax = 10, working memory = 10 (as indicated on the scoring sheet, see Supplementary Figure 2B below).

Error categories are signified by the shape of the checkboxes next to each potential error: diamond = motor speech error; circle = phonological error; square = semantic knowledge error; hexagon = syntactic error, star = working memory error. On each page, count the number of checkboxes of each shape that contain an ‘X’, and note the total in the corresponding shape at the bottom of the page (see Supplementary Figure 2A). Pay attention to any instructions to multiply the X (indicated with a note underneath relevant checkboxes; see Supplementary Figure 1). The ‘Totals’ at the bottom of each page should then be transferred to the relevant row in the scoresheet at the back of the booklet (see Supplementary Figure 2B).

**Overall score:** An overall score out of 100 can be calculated by summing the total number of errors made across the domains, and subtracting this number from 100 (see Supplementary Figure 2B).

**
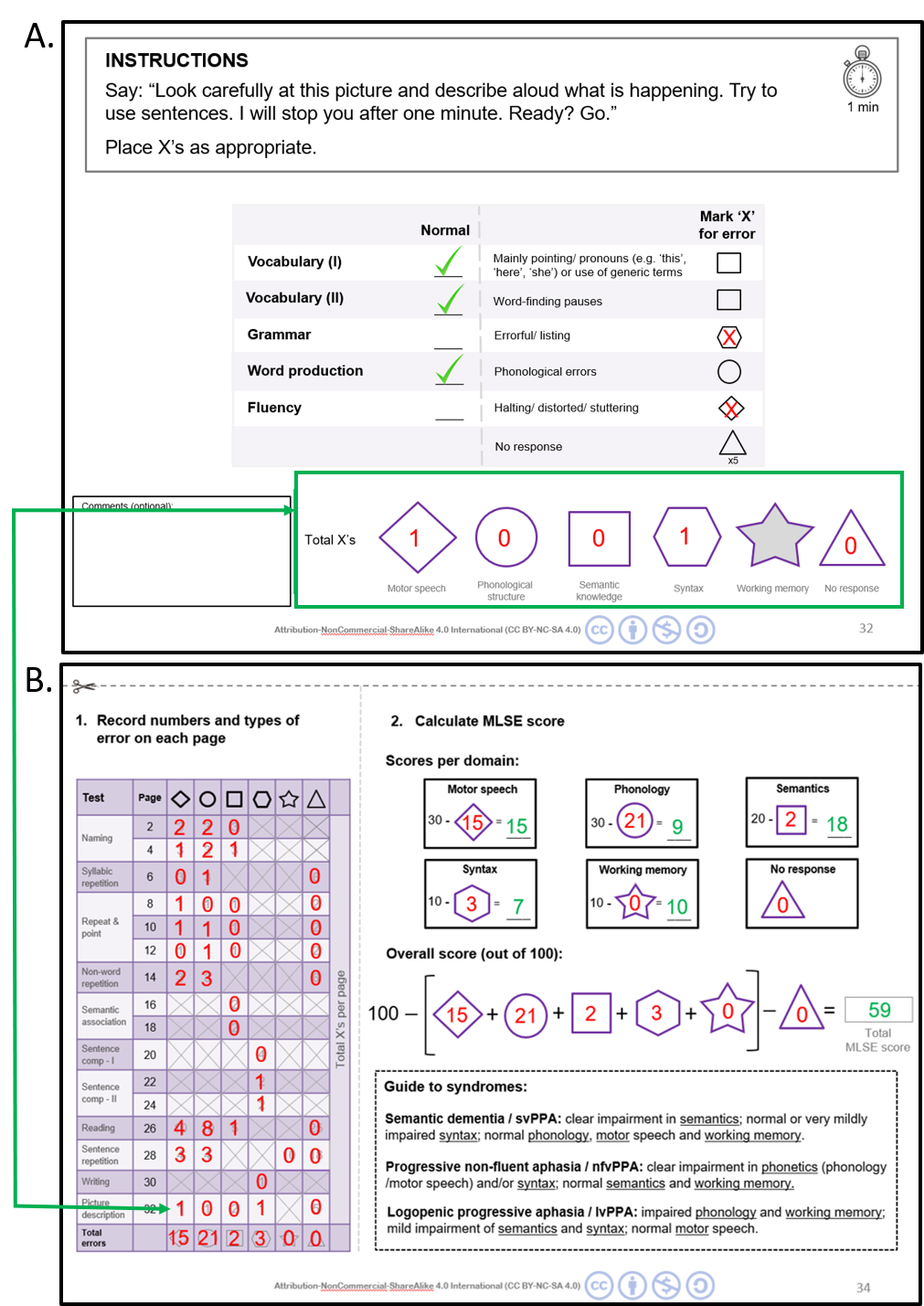
**

**Supplementary Figure 2.** Example scoring for the (A) picture description task, and (B) completed score sheet for the entire test

# How to score if a participant has severe speech production deficits

While the MLSE is not designed for use with people who have severe speech production deficits, it can still provide useful information about an individual’s linguistic ability beyond speech production. If the test is to be used in this way, the following modifications to the administration/scoring are suggested:

1. A participant may provide written responses for the Naming task, but if they need to do so “Distorted” should be ticked.
2. Repetition and reading tasks can be skipped and scored as “No Response”.
3. A participant may provide a written description of the picture for the Picture Description task in order to assess vocabulary and grammar, but the “Motor Speech” domain (Fluency) must be marked as an error.

In such cases, scoring according to five linguistic domains would no longer be possible but overall scores can provide useful information about severity of impairment.

# Guidance to printing in booklet format

When printing the document, printing preference opens a dialogue box which allows you to configure printer settings for page layout (Supplementary Figure 3). Under page sizing and handling, please select ‘*Booklet’, ‘Portrait’* **and** check the ‘*Auto-rotate pages within each sheet*’ box’ (as indicated by the arrow in Supplementary Figure 3).


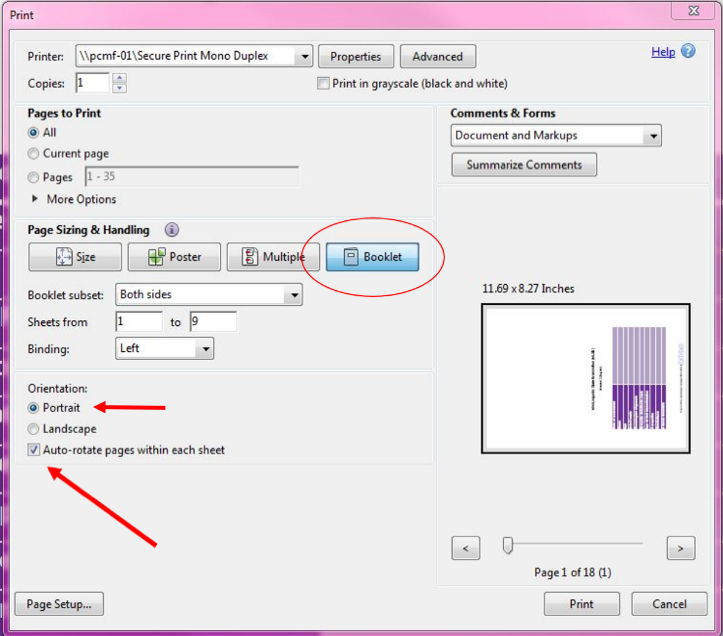


**Supplementary Figure 3.** Printing preference dialogue

**Supplementary Table 10**: Feature rankings which expressed the accuracy across partitions of training data, comparing all possible number and combination of domains for classifying lvPPA, nfvPPA, svPPA and control groups within the unseen testing data.

| Features |  | **lvPPA** | **nfvPPA** | **svPPA** | **Controls** |
| --- | --- | --- | --- | --- | --- |
| **5** | Selected features | M, SY, WM, P, S | M, P, WM, S, SY | M, S, SY, WM, P | M, P, S, SY, WM |
|  | Accuracy (F1) | 0.824 | 0.786 | 0.933 | 1.000 |
|  | Sensitivity | 0.883 | 0.866 | 1.000 | 1.000 |
|  | Specificity | 0.995 | 0.951 | 0.980 | 1.000 |
|  | **Balanced accuracy** | **0.919** | **0.909** | **0.990** | **1.000** |
| **4** | Selected features | M, P, WM, S | M, P, WM, SY | M, P, S, WM | M, P, SY, S |
|  | Accuracy (F1) | 0.891 | 0.826 | 0.933 | 1.000 |
|  | Sensitivity | 0.883 | 0.933 | 1.000 | 1.000 |
|  | Specificity | 0.977 | 0.951 | 0.980 | 1.000 |
|  | **Balanced accuracy** | **0.931** | **0.943** | **0.990** | **1.000** |
| **3** | Selected features | SY, WM, M | M, P, SY | M, S, SY | WM, SY, S |
|  | Accuracy (F1) | 0.853 | 0.833 | 0.866 | 1.000 |
|  | Sensitivity | 0.933 | 0.900 | 0.900 | 1.000 |
|  | Specificity | 0.955 | 0.955 | 0.980 | 1.000 |
|  | **Balanced accuracy** | **0.944** | **0.927** | **0.940** | **1.000** |
| **2** | Selected features | WM, SY | SY, WM | S, SY | P, S |
|  | Accuracy (F1) | 0.893 | 0.866 | 0.866 | 0.914 |
|  | Sensitivity | 0.866 | 0.900 | 0.900 | 0.950 |
|  | Specificity | 0.975 | 0.971 | 0.980 | 1.000 |
|  | **Balanced accuracy** | **0.920** | **0.935** | **0.940** | **0.975** |
| **1** | Selected features | WM | M | S | S |
|  | Accuracy (F1) | 0.707 | 0.766 | 0.700 | 0.892 |
|  | Sensitivity | 0.746 | 0.900 | 0.700 | 0.910 |
|  | Specificity | 0.935 | 0.932 | 0.955 | 0.946 |
|  | **Balanced accuracy** | **0.840** | **0.916** | **0.827** | **0.928** |

M; Motor Speech, P; Phonology, S; Semantics, SY; Syntax, WM; Working memory


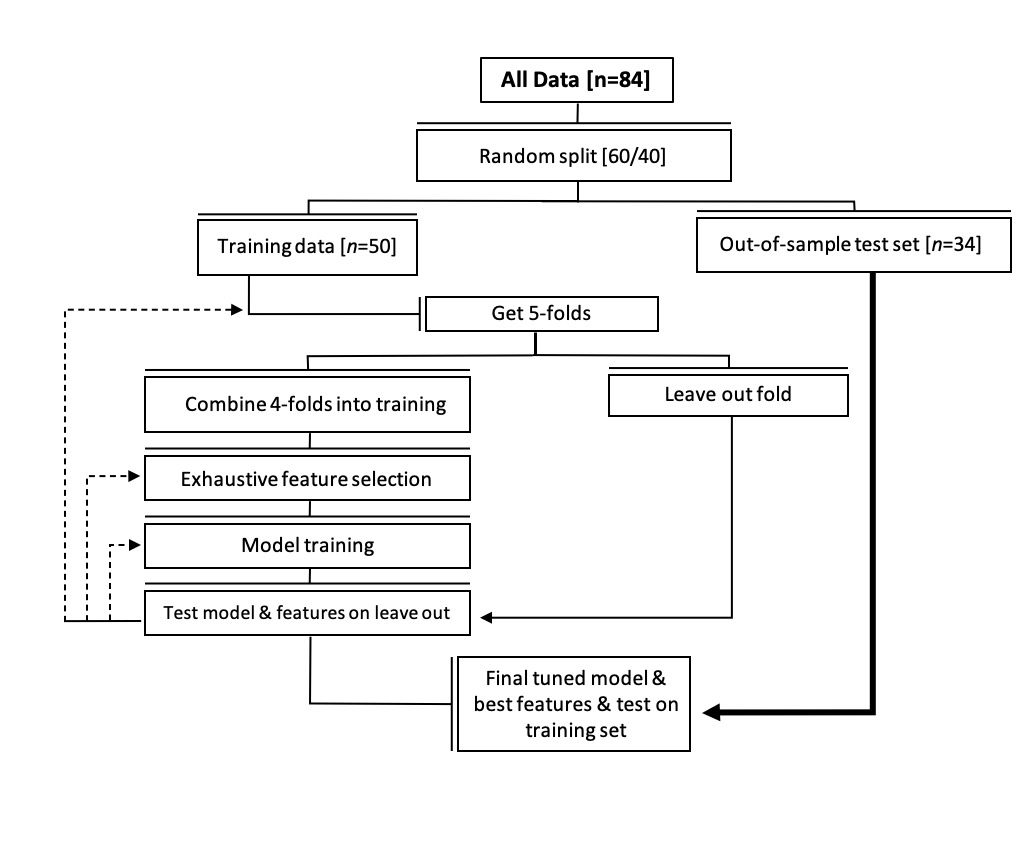


**Supplementary Figure 4.** Schematic representation of the methodology used to generate the random forest classification model
